# Human iPSC-derived mesoangioblasts, like their tissue-derived counterparts, suppress T cell proliferation through IDO- and PGE-2-dependent pathways

**DOI:** 10.12688/f1000research.2-24.v1

**Published:** 2013-01-25

**Authors:** Ou Li, Karen English, Rossana Tonlorenzi, Giulio Cossu, Francesco Saverio Tedesco, Kathryn J Wood

**Affiliations:** 1 Transplantation Research Immunology Group, Nuffield Department of Surgical Sciences, University of Oxford, Oxford, UK; 2 Cellular Immunology Group, Institute of Immunology, National University of Ireland Maynooth, Co. Kildare, Ireland; 3 Division of Regenerative Medicine, Stem Cells and Gene Therapy, San Raffaele Scientific Institute, Milan, Italy; 4 Department of Cell and Developmental Biology and Centre for Stem Cells and Regenerative Medicine, University College London, London, UK

## Abstract

Human mesoangioblasts are currently in a phase I/II clinical trial for the treatment of patients with Duchenne muscular dystrophy. However, limitations associated with the finite life span of these cells combined with the significant numbers of mesoangioblasts required to treat all of the skeletal muscles in these patients restricts their therapeutic potential. Induced pluripotent stem cell (iPSC)-derived mesoangioblasts may provide the solution to this problem. Although, the idea of using iPSC-derived cell therapies has been proposed for quite some time, our understanding of how the immune system interacts with these cells is inadequate. Herein, we show that iPSC-derived mesoangioblasts (HIDEMs) from healthy donors and, importantly, limb-girdle muscular dystrophy 2D patients exert immunosuppressive effects on T cell proliferation.  Interferon gamma (IFN-γ) and tumour necrosis factor alpha (TNF-α) play crucial roles in the initial activation of HIDEMs and importantly indoleamine 2,3 dioxygenase (IDO) and prostaglandin E2 (PGE-2) were identified as key mechanisms involved in HIDEM suppression of T cell proliferation. Together with recent studies confirming the myogenic function and regenerative potential of these cells, we suggest that HIDEMs could provide an unlimited alternative source for mesoangioblast-based therapies.

## Introduction

Preclinical models of muscular dystrophy in both mouse (α-sarcoglycan-null, dysferlin-null and mdx) ^1– 4^ and dog (golden retriever muscular dystrophy) ^2^ provided evidence that mesoangioblasts have the capacity to functionally ameliorate the dystrophic phenotype. Based on these data, human allogeneic HLA-identical mesoangioblasts are in a phase I/II clinical trial for the treatment of paediatric patients affected by Duchenne muscular dystrophy ( DMD; EudraCT no. 2011-000176-33). However, human mesoangioblasts have a finite lifespan in culture and the need for large cell numbers to treat each patient may impact the therapeutic efficacy of this approach, especially if this treatment should be extended to adults. To address and overcome these issues, the concept of deriving patient-specific induced pluripotent stem cells (iPSCs) that could serve as a platform for the genetic correction of autologous cell therapies in the future is being explored ^5^.

Developments in the generation of iPSCs have advanced significantly in the past 6 years ^6, 7^; however, concerns related to potential tumorigenicity, incomplete and inefficient terminal differentiation ^8^, as well as immunogenicity of iPSCs ^9^ and in particular their differentiated progeny remains to be fully elucidated. Immune rejection represents a significant hurdle not just for allogeneic cell therapies, but for all cell therapy approaches, and questions surrounding the potential immunogenicity of iPSCs and their derivatives have been largely overlooked. A recent study provides evidence that syngeneic mouse iPSCs are more immunogenic than their embryonic stem cell (ESC) counterparts ^9^. Immune responses against the integrated viral vectors ^9^ as well as allogeneic ^10^ and mutated mitochondrial genome after long term culture ^11^ of iPSCs have been reported. Moreover, rejection of syngeneic mouse iPSCs following transplantation *in vivo* into mice ^9^ highlights the fact that even autologous iPSCs can be recognised by the immune system and will be subject to standard rejection mechanisms ^12, 13^. Therefore, characterising the interactions between iPSC-derived differentiated cells and immune cells and determining whether or not these cells are recognised and rejected by the immune system is critically important.

To explore these possibilities, we utilised a novel protocol to derive mesoangioblast-like cells (human iPSC-derived mesoangioblasts: HIDEMs) initially from healthy iPSCs and subsequently from iPSCs reprogrammed from skeletal muscle cells of Limb-Girdle Muscular Dystrophy, Type 2D (LGMD2D) patients ^14^. These cells, as expected, exert similar myogenic potential as normal mesoangioblasts, which makes them candidates for future clinical application. Here, we examined the effect of HIDEMs derived from both healthy donors and LGMD2D patients on immune cells *in vitro* and compared them with conventionally generated mesoangioblasts from healthy donors. Importantly, we demonstrate that HIDEMs from both sources do not induce, but rather suppress mitogen-driven T cell proliferation *in vitro*. Moreover, consistent with our findings for adult skeletal muscle-derived human mesoangioblasts ^15^, HIDEMs mediate their immune suppressive effects through indoleamine 2,3 dioxygenase (IDO) and prostaglandin E2 (PGE-2).

## Materials & methods

### Cell culture

Human mesoangioblasts were isolated from adult skeletal muscle as previously described ^16^. The HIDEM lines utilized in this study were generated from human iPSCs derived from either healthy donors (HIDEMs #1) or patients affected by limb-girdle muscular dystrophy 2D (LGMD2D Pt.3 HIDEMs) and are described in detail in a recent publication ^14^. Both cell types were cultured in Iscove’s Modified Dulbecco’s Medium (IMDM; PAA) containing 10% FBS (Gibco), 2 mM glutamine (PAA), 0.1 mM β-mercaptoethanol (Sigma-Aldrich), 1% NEAA (PAA), 5 ng/ml human bFGF (Peprotech), 100 IU ml ^–1^ penicillin (PAA), 100 mg/ml ^–1^ streptomycin (PAA), 1% insulin transferrin selenium X (ITSX) supplement (Gibco), 0.5 μM oleic and linoleic acids (Sigma-Aldrich), 1.5 mM Fe++ (Iron ^10^ chloride tetrahydrate, Sigma; or Fer-In-Sol, Mead Johnson), 0.12 mM Fe+++ (Iron(III) nitrate nona- hydrate, Sigma; or Ferlixit, Aventis). Myogenic differentiation of mesoangioblasts and HIDEMs was performed as previously described ^14, 16^.

Human peripheral blood mononuclear cells (PBMCs) were isolated from buffy coats. PBMCs from 4 donors were used in this study. All PBMC cultures were carried out in RPMI 1640 (Sigma-Aldrich) supplemented with 10% FBS (Gibco), 1% v/v L-glutamine (PAA), 0.05mM β-mercaptoethanol (Sigma-Aldrich) and 100 IU ml ^–1^ penicillin (PAA) and 100 mg/ml ^–1^ streptomycin (PAA).

### T cell proliferation assays

Proliferation of PBMCs was measured using the 5,6 carboxyfluorescein diacetate succinimidyl ester (CFSE) dilution assay. PBMCs were labelled with 10µM CFSE (Invitrogen) in warm phosphate buffered saline (PBS) at room temperature. After 10 minutes, cells were washed in ice cold PBS. CFSE-abelled PBMCs (2.5 × 10 ^5^/ml) were then seeded into 96-well round bottom plates with or without human anti-CD3/CD28 beads (Dynal-Life Technologies, UK) (0.5 × 10 ^5^/ml) in complete RPMI 1640 (Sigma-Aldrich). For suppressor assays, mesoangioblasts/HIDEMs were seeded overnight in 96-well round bottom plates in mesoangioblast/HIDEM cell culture medium. The medium was then replaced with complete RPMI 1640 containing PBMC and anti CD3/CD28 beads where appropriate. Cells were harvested on day 6 and CFSE dilution was analysed by flow cytometry (FACSCanto ^TM^ II, BD Bioscience, UK). Actual numbers of CFSE-divided cells were calculated using counting beads (BD Biosciences, UK).

### Flow cytometric analysis

Mesoangioblasts and HIDEMs were characterised for the following phenotypic surface markers: HLA-ABC, HLA-DR (BD Biosciences), CD105, CD73, CD49b (eBioscience) and CD146 (Biocytex). In addition, mesoangioblast expression of CD40 (BD Biosciences) and PD-L1 (eBioscience) as well as the HLA molecules were analysed before and after stimulation (24h) with the pro-inflam matory cytokines interferon gamma (IFN-γ), tumour necrosis factor alpha (TNF-α) or interleukin 1-beta (IL-1β) (Peprotech, UK). The effect of mesoangioblast on the expression of early activation markers on T cells was analysed by flow cytometry. Cells were harvested on day 3, 4, 5 and 6 and stained with anti-CD3, anti-CD25, anti-CD69 and 7AAD (eBioscience).

### Neutralising and blocking studies

Proliferation assays in the form of CFSE dilution were carried out as described above in the presence or absence of neutralising antibodies to IFN-γ (R&D Systems), TNF-α (eBioscience), isotype control (R&D Systems) or recombinant IL-1 receptor antagonist (IL-1RA) (eBioscience) at concentrations of 0.5, 1.0 and 2.0 ug/ml. Inhibitors of IDO-1-Methyl-L-tryptophan (1MT) (Sigma-Aldrich) (0.5 mM), and of Cox-2: NS-398 (Calbiochem, UK) (1.0 uM) were used in this study.

### Statistical methods

Data were analysed using the statistical software Prism (version 5; GraphPad Software) and are reported as mean +/- standard error (SE). The unpaired Student *t* test was performed to compare 2 mean values. Otherwise, data were analysed using a two-way ANOVA with Bonferonni’s post-test and *p* values <0.05 were considered statistically significant.

## Results

### HIDEMs share the same immuno-phenotype and myogenic potential with mesoangioblasts

**Figure 1.**
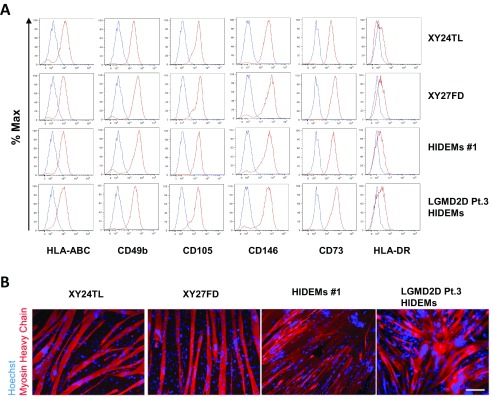
HIDEM and mesoangioblast surface marker phenotype and differentiation capabilities. Healthy donor HIDEM line #1 and LGMD2D HIDEMs from patient no. 3 (HIDEMs Pt.3) and mesoangioblasts XY24TL and XY27FD were analysed for their surface marker expression of HLA-ABC, HLA-DR, CD105, CD73, CD49b and CD146 ( **A**). Mesoangioblasts were stained with fluorochrome-labelled antibodies against the above markers (shown in red) as well as isotype matched control antibodies (shown in blue) followed by flow cytometric analysis. All four cell lines were also induced for myogenic differentiation followed by immunostaining of myocyte/myotube marker Myosin Heavy Chain (red). Cell nuclei were counter stained with Hoechst (blue). Scale bar = 100 µm ( **B**).

Cultured HIDEM lines, HIDEMs #1 and LGMD2D Pt.3 HIDEMs, together with mesoangioblast lines XY24TL and XY27FD were characterised for their surface expression of pericyte makers CD73, CD105, CD49b, CD146, and HLA molecule HLA-ABC and HLA-DR. HIDEM lines and mesoangioblast lines expressed all of the aforementioned pericyte markers as well as HLA-ABC and low level expression of HLA-DR, indicating that these cells share the same phenotype ( Figure 1A). HIDEM cells were also characterized for their myogenic potential with differentiation into skeletal myocytes/myotubes. Myogenic differentiation was observed in HIDEM lines, like in mesoangioblasts, by immunostaining for the striated muscle specific marker myosin heavy chain ( Figure 1B), with the only difference that HIDEM terminal differentiation needed the expression of the myogenic regulator MyoD, as recently reported ^14^.

### Mesoangioblasts do not provoke T cell proliferation *in vitro*, even in the presence of inflammatory mediators

**Figure 2.**
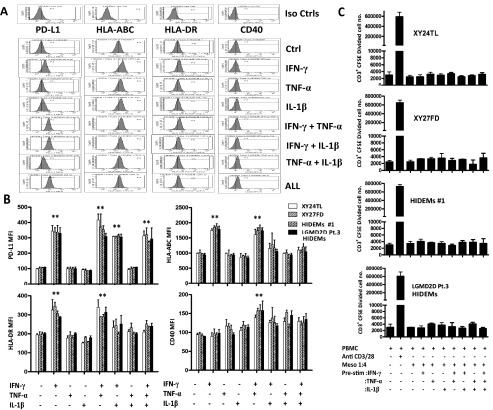
HIDEMs and mesoangioblasts fail to induce T cell proliferation *in vitro*. HIDEMs and mesoangioblasts were stimulated with IFN-γ, TNF-α or IL-1β (20ng/ml) for 24h. Cells were trypsinized and washed, followed by surface staining for HLA-ABC, HLA-DR, CD40, PD-L1 or fluorochrome matched isotype controls and analysis by flow cytometry. Representative histograms of HIDEMs #1 are presented here ( **A**). Data are represented as mean (+/- SE) median fluorescence intensity of the markers examined over 4 replicates. **p<0.01 ( **B**). CFSE-labelled PBMCs were stimulated with anti CD3/CD28 beads (PBMC+B) as a positive control. Non-stimulated or cytokine stimulated HIDEMs/mesoangioblasts (ratio 1:4) were co-cultured with PBMC for 6 days. CD3 ^+^ CFSE labelled 7AAD ^-^ cells were enumerated using counting beads ( **C**). Experiments were carried out in duplicates. n=4.

It has been shown that allogeneic stem cells enjoy some level of immune privilege ^17– 21^. However, the general consensus is that differentiated cells tend to be more immunogenic than undifferentiated stem cells as demonstrated by studies using embryonic stem cells ^18, 22^. Thus, it was important to compare the immunogenicity of HIDEMs with that of mesoangioblasts derived from conventional adult muscle under basal (see above) or simulated inflammatory conditions. Following stimulation with the pro-inflammatory cytokines IFN-γ, TNF-α or IL-1β or a combination of these soluble mediators, expression of HLA-ABC, HLA-DR, PD-L1 were increased after stimulation with IFN-γ, but not TNF-α or IL-1β ( Figure 2A and 2B). Stimulation of HIDEMs and mesoangioblasts with IFN-γ and TNF-α or combinations of these cytokines induced low-level expression of CD40 ( Figure 2A and 2B).

Raw data for Figure 2B: Change of surface marker expression of Mesoangioblasts/HIDEMs upon pro-inflammatory stimulation HIDEMs and mesoangioblasts were stimulated with IFN-γ, TNF-α or IL-1β (20ng/ml) for 24h. Cells were trypsinized and washed, followed by surface staining for HLA-ABC, HLA-DR, CD40, PD-L1 or fluorochrome matched isotype controls and analysis by flow cytometry. Experiments were carried out in duplicates. n=4. Median fluorescence intensities of the markers were examined, and were shown as Mean ± SE.

Raw data for Figure 2C: HIDEMs and mesoangioblasts fail to induce T cell proliferation in vitro CFSE labelled PBMCs were stimulated with anti CD3/CD28 beads (PBMC+B) as a positive control. HIDEMs and mesoangioblasts were stimulated with IFN-γ, TNF-α or IL-1β (20ng/ml) for 24h. Non-stimulated or cytokine stimulated HIDEMs/mesoangioblasts (ratio 1:4) were then co-cultured with PBMC for 6 days. CD3+ CFSE labelled 7AAD- cells were enumerated using flow cytometry and counting beads. Experiments were carried out in duplicates. n=4.

To examine their immunogenicity, HIDEMs and mesoangioblasts cultured under basal conditions or pre-stimulated with the pro-inflammatory cytokines IFN-γ, TNF-α or IL-1β were co-cultured with allogeneic CFSE-labelled PBMCs. Neither HIDEMs nor mesoangioblasts induced CD3 ^+^ T cell proliferation ( Figure 2C) *in vitro* after 6 days of co-culture. Furthermore, exposure of HIDEMs to pro-inflammatory cytokines did not alter this lack of T cell response ( Figure 2C). Thus, T cells were unresponsive to allogeneic HIDEMs even after pre-stimulation with pro-inflammatory cytokines.

### Both HIDEMs and mesoangioblasts suppress T cell proliferation in a dose-dependent manner, but do not interfere with T cell activation

**Figure 3.**
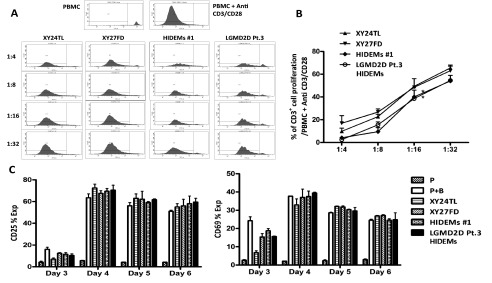
Mesoangioblasts suppress T cell proliferation in a dose-dependent manner without interfering with T cell activation. CFSE-labelled PBMCs (5 × 10 ^4^/well) were stimulated with anti CD3/CD28 beads (1 × 10 ^4^/well) (P+B) in the presence or absence of HIDEMs/mesoangioblasts at decreasing ratios (HIDEM/mesoangioblast:PBMC). On day 6, cells were harvested and stained with anti-CD3 and 7AAD, and analysed by flow cytometry. CFSE dilution was analysed on gated CD3 ^+^7AAD ^-^ cells. Representative histograms showing CD3 ^+^7AAD ^-^ cells with CFSE dilutions after co-culture with HIDEM/mesoangioblast ( **A**). The percentage of CD3 ^+^CFSE dividing cells calculated for each group and compared to the positive control (P+B). *p<0.05 ( **B**). Cells were harvested on day 3, 4, 5 or 6 and analysed for CFSE dilution and expression of CD25 and CD69. The number of CD3 ^+^7AAD ^-^ cells expressing CD25 or CD69 using counting beads and the % of CD25 ^+^ and CD69 ^+^ cells were calculated from the data ( **C**). Experiments were carried out in duplicates. n=2. Unpaired *t* tests were used for statistical analysis.

Human mesoangioblasts have the capacity to modulate T cell proliferation *in vitro* ^15^. Here, we sought to determine whether HIDEMs could exert immune suppressive effects. All 4 cell lines potently suppressed CD3 ^+^ T cell proliferation *in vitro* in a dose-dependent manner ( Figure 3A and 3B). Interestingly, HIDEMs from both healthy donors and patients showed greater suppressive potency compared to mesoangioblasts at the same cell:PBMC ratio. We therefore hypothesised that HIDEMs may alter T cell activation. The effect of HIDEMs/mesoangioblasts on the expression of early T cell activation markers (CD25 and CD69), was directly examined on PBMCs stimulated with anti-CD3/CD28 beads in the presence or absence of HIDEMs or mesoangioblasts. The percentage of T cells expressing CD25 and CD69 increased significantly between days 3 and 4 after stimulation as expected, and the presence of HIDEMs or mesoangioblasts inhibited the expression of CD69 on day 3 but had no effect on days 4–6 ( Figure 3C). Expression of CD25 was not significantly affected by HIDEMs or mesoangioblasts at any time point examined ( Figure 3C). Thus, although HIDEMs exhibited more potent activity with respect to inhibition of T cell proliferation, they did not differentially impact T cell activation.

Raw data for Figure 3B: Mesoangioblasts and HIDEMs suppress T cell proliferation in a dose dependent manner CFSE labelled PBMCs (5 x 104/well) were stimulated with anti CD3/CD28 beads (1 x 104/well) (P+B) in the presence or absence of HIDEMs/mesoangioblasts at decreasing ratios (HIDEM/mesoangioblast:PBMC). On day 6 cells were harvested and stained with anti-CD3 antibody and 7AAD, and analysed by flow cytometry. CFSE dilution was analysed on gated CD3+ 7AAD- cells. The percentage of CD3+CFSE dividing cells was calculated for each group and compared to the positive control (P+B), followed by plotting against HIDEM/mesoangioblast:PBMC ratios. Experiments were carried out in duplicates. n=2

Raw data for Figure 3C: Mesoangioblasts and HIDEMs do not interfer with T cell activation CFSE labelled PBMCs (5 x 104/well) were stimulated with anti CD3/CD28 beads (1 x 104/well) (P+B) in the presence or absence of HIDEMs/mesoangioblasts at HIDEM/mesoangioblast:PBMC = 1:4 ratio. Cells were harvested on day 3, 4, 5 or 6 and analysed for CFSE dilution and expression of CD25 and CD69. The number of CD3+7AAD- cells expressing CD25 or CD69 using counting beads and the % of CD25+ and CD69+ cells were calculated from the data. Experiments were carried out in duplicates. n=2.

### IFN-γ and TNF-α play important roles in the suppression of T cell proliferation by HIDEMs

**Figure 4.**
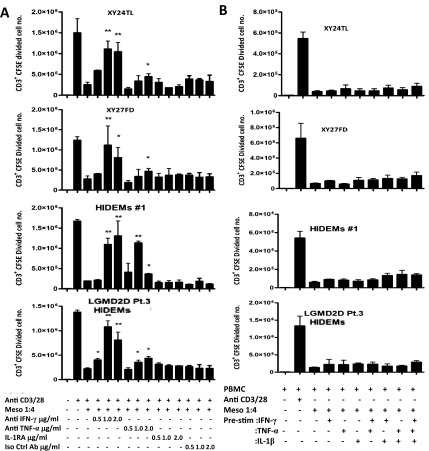
IFN-γ and TNF-α play important roles in HIDEM and mesoangioblast suppression of T cell proliferation. CFSE-labelled PBMCs were stimulated with anti-CD3/CD28 beads in the presence of HIDEMs/mesoangioblasts (1:4) and neutralising antibodies against IFN-γ and TNF-α or irrelevant isotype control antibody (0.5, 1.0 and 2.0 µg/ml) or recombinant IL-1RA (0.5, 1.0 and 2.0 µg/ml). Cells were harvested on day 6 and stained with anti-CD3 and 7AAD. After gating on CD3 ^+^7AAD ^-^ the number of CFSE diluting cells were enumerated using counting beads. Data are represented as mean CD3 ^+^ CFSE diluted cell number +/- SE. *p<0.05, **p<0.01 ( **A**). HIDEMs/mesoangioblasts were left untreated or were stimulated with IFN-γ, TNF-α or IL-1β (20ng/ml) for 24h before setting up co-cultures with CFSE labelled PBMCs. As a positive control, PBMCs were stimulated with anti CD3/CD28 beads. After 6 days cells were harvested and surface stained for CD3 and 7AAD before analysis of CFSE dilution. CD3 ^+^CFSE diluted cell numbers were calculated using counting beads as before ( **B**). Experiments were carried out in duplicates. n=4.

As IFN-γ, TNF-α and IL-1β have been identified as key cytokines required for the activation of mesoangioblast immunosuppressive function *in vitro* ^15^, we examined the importance of these pro-inflammatory cytokines in the modulation of T cell proliferation by HIDEMs. Neutralising antibodies against IFN-γ at concentrations of 1.0 and 2.0 ug/ml significantly abolished the inhibitory effect mediated by HIDEMs and mesoangioblasts ( Figure 4A). A similar, but weaker effect was observed in the presence of a neutralising antibody against TNF-α (2.0 ug/ml) for both cell types. Notably, a lower concentration of anti-TNF-α (1.0 ug/ml) partially restored T cell proliferation in the presence of HIDEMs, but not mesoangioblasts, suggesting that TNF-α may play a more important role in the suppressive function of HIDEMs compared to mesoangioblasts ( Figure 4A). The addition of IL-1 receptor antagonist (IL-1RA) at concentrations of 0.5, 1.0 or 2.0 ug/ml did not restore T cell proliferation in this case.

Raw data for Figure 4A: Neutralising antibodies against IFN-γ and TNF-α reduce the immunosuppressive capacity of Mesoangioblasts/HIDEMs CFSE labelled PBMCs were stimulated with anti-CD3/CD28 beads in the presence of HIDEMs/mesoangioblasts (1:4) and neutralising antibodies against IFN-γ and TNF-α or irrelevant isotype control antibody (0.5, 1.0 and 2.0 µg/ml) or recombinant IL-1RA (0.5, 1.0 and 2.0 µg/ml). Cells were harvested on day 6 and stained with anti-CD3 and 7AAD. After gating on CD3+7AAD- the number of CFSE diluting cells were enumerated using counting beads. Experiments were carried out in duplicates. n=4.

Raw data for Figure 4B: Pre-stimulation with IFN-γ, TNF-α and IL-1β does not enhance the immunosuppressive effect of Mesoangioblasts/HIDEMs HIDEMs/mesoangioblasts were left untreated or were stimulated with IFN-γ, TNF-α or IL-1β (20ng/ml) for 24h before setting up co-cultures with CFSE labelled PBMC and anti CD3/CD28 beads. After 6 days cells were harvested and surface stained for CD3 and 7AAD before analysis of CFSE dilution. CD3+CFSE diluted cell numbers were calculated using counting beads as before. Experiments were carried out in duplicates. n=4.

Our previous study showed that pre-stimulation with IFN-γ, TNF-α or IL-1β does not enhance or abrogate mesoangioblast suppression of T cell proliferation *in vitro*. To test if this holds true for HIDEMs, cells were then pre-treated with IFN-γ, TNF-α, IL-1β, or combinations of these cytokines for 24h before adding them to CFSE dilution assays to examine their immunosuppressive capacity. Pre-stimulation did not promote the suppressor capacity of HIDEMs or mesoangioblasts, compared to non-stimulated controls ( Figure 4B). However, there was no significant loss of suppressor capacity by HIDEMs following stimulation with pro-inflammatory cytokines, similar to that observed for mesoangioblasts.

### HIDEMs utilise both IDO and PGE-2 in the suppression of T cell proliferation

**Figure 5.**
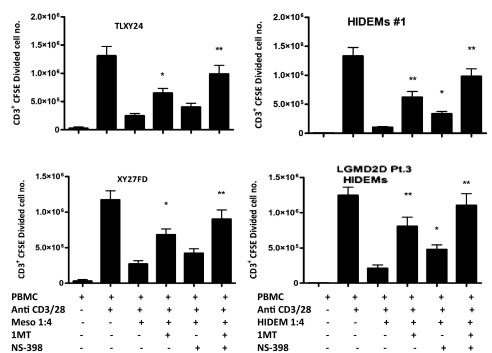
IDO and PGE-2 are involved in HIDEMs suppression of T cell proliferation. CFSE-labelled PBMCs were stimulated with anti CD3/CD28 beads as before in the presence of HIDEMs/mesoangioblasts and inhibitors of IDO and Cox-2, (1-Methyl-L-trypyophan (1MT) (0.5 mM) and NS-398 (1.0 uM) respectively, or both. On day 6 cells were harvested and stained with anti-CD3 and 7AAD. Cells were gated on live CD3 ^+^ populations and analysed for CFSE dilution and the numbers of cells undergoing CFSE dilution were enumerated using counting beads, *p<0.05, **p<0.01. Experiments were carried out in duplicates. n=4.

IDO and PGE-2 have been identified as key mechanisms in the modulation of T cell proliferation by mesoangioblasts. To test the hypothesis that IDO and PGE-2 are key pathways used by HIDEMs for modulating T cell proliferation, 1-Methyl-L-tryptophan (1MT) (an inhibitor of IDO) or NS-398 (1.0 µM) (a specific Cox-2 inhibitor) were added to T cell cultures containing HIDEMs or mesoangioblasts. Both inhibitors partially, but significantly, restored proliferation, with 1MT having the most potent effect. Addition of both inhibitors showed an additive effect ( Figure 5). Restoration of proliferation mediated by the addition of NS-398 was significant in cultures containing HIDEMs, but not mesoangioblasts ( Figure 5).

Raw data for Figure 5: The presence of IDO and PGE-2 inhibitors reduce the suppression of T cell proliferation by Mesoangioblasts/HIDEMs CFSE labelled PBMCs were stimulated with anti CD3/CD28 beads as before in the presence of HIDEMs/mesoangioblasts and inhibitors of IDO and Cox-2, (1-Methyl-L-trypyophan (1MT) (0.5mM) and NS-398 (1.0 uM) respectively, or both. On day 6 cells were harvested and stained with anti-CD3 and 7AAD. Cells were gated on live CD3+ populations and analysed for CFSE dilution and the numbers of cells undergoing CFSE dilution were enumerated using counting beads. Experiments were carried out in duplicates. n=4.

## Discussion

The advent and unwavering development of iPSC technology makes autologous cell therapy and personalised gene therapy a realistic possibility for many degenerative and genetic diseases like muscular dystrophies. Nevertheless, a number of reports have raised concerns on the overlooked possibility that iPSCs and their derivatives may be targeted by the immune system for various reasons ^9, 10, 23^. The recent new development of human iPSC-derived mesoangioblasts may provide an unlimited source for mesoangioblast therapy for DMD, but understanding the immunogenicity of these cells is critically important for their successful application.

In the current study, we demonstrate that HIDEMs behave in a very similar manner to mesoangioblasts with regard to expression of HLA and co-stimulatory molecules, suppressive potency and mechanisms of action. We showed that HIDEMs are comparable to mesoangioblasts in their surface marker expression and that both cell populations respond to stimulation with pro-inflammatory cytokines consistently with increased expression of HLA-ABC, HLA-DR, PD-L1 and low level expression of CD40. Importantly, HIDEMs suppress T cell proliferation driven by anti-CD3/CD28 stimulation in a similar dose-dependent manner as that of mesoangioblasts, but with increased potency. Comparable to mesoangioblasts, HIDEMs did not alter the expression of early activation markers CD25 or CD69 on CD3 ^+^ T cells, a phenomenon also seen with immune-suppressive mesenchymal stromal cells (MSC) ^24^.

An activation step involving IFN-γ or TNF-α was a prerequisite for HIDEM suppression of T cell proliferation as neutralisation of either cytokine significantly abolished the inhibitory effect. IDO and PGE-2 were identified as key mechanisms of action involved in HIDEM suppression of T cell proliferation. Overall, the immunogenicity and immunosuppressive capacity of HIDEMs was almost indistinguishable from that of their conventionally derived counterpart, mesoangioblasts ^15^. The only minor differences between these two cell populations were the increased immunosuppressive potency and sensitivity of HIDEMs to TNF-α and to the specific Cox-2 inhibitor NS-398. Perhaps these three observations are linked and suggest that HIDEMs are more receptive to TNF-α, leading to the induction of enhanced levels of PGE-2 facilitating a more potent inhibition of T cell proliferation. Importantly, this study demonstrates that iPSC derived cells (HIDEMs) display immunomodulatory functions equivalent to those of their tissue-derived counterpart, mesoangioblasts.

Gene therapy is an appealing approach to treat many hereditary diseases such as DMD, and adeno-associated virus-based gene therapy trials showed promising results for LGMD2D patients ^25^. However, problems associated with immunity to dystrophin and subsequent loss of transgene expression might limit the successful application of this approach ^26, 27^. The use of retro-/lenti-viral vector-based techniques for the generation of iPSCs and for gene correction of their progeny means that cells derived from iPSCs using these technologies could face the same challenge. Nevertheless, HIDEMs have been derived in a complete viral integration-free setting ^14^ and, although we have not examined their immunomodulatory function *in vivo*, the proven therapeutic value of iPSC-derived mesoangioblasts, combined with their potential immune suppressive characteristics, means that HIDEMs are well placed to overcome some of the challenges facing iPSC-derived cell therapies.
