# Supplementary material for: Human iPSC-derived mesoangioblasts, like their tissue-derived counterparts, suppress T cell proliferation through IDO- and PGE-2-dependent pathways
Source: F1000Res. 2013 Jan 25;2:24. [Version 1] doi: 10.12688/f1000research.2-24.v1 (PMC3968899; doi:10.12688/f1000research.2-24.v1)
Supplement: Raw data for Figure 2B: Change of surface marker expression of Mesoangioblasts/HIDEMs upon pro-inflammatory stimulation — HIDEMs and mesoangioblasts were stimulated with IFN-γ, TNF-α or IL-1β (20ng/ml) for 24h. Cells were trypsinized and washed, followed by surface staining for HLA-ABC, HLA-DR, CD40, PD-L1 or fluorochrome matched isotype controls and analysis by flow cytometry. Experiments were carried out in duplicates. n=4. Median fluorescence intensities of the markers were examined, and were shown as Mean ± SE. [file f1000research-2-1191-s0000.tgz › CD40_MFI.pdf]

Table format: **Grouped**		**Group A**							
		Data Set-A							
		**A:Y1**	**A:Y2**	**A:Y3**	**A:Y4**	**A:Y5**	**A:Y6**	**A:Y7**	**A:Y8**
**1**									
**2**									
**3**									
**4**									
**5**									
**6**									
**7**									
**8**									

	Group B								
	Data Set-B								
	**B:Y1**	**B:Y2**	**B:Y3**	**B:Y4**	**B:Y5**	**B:Y6**	**B:Y7**	**B:Y8**	**C:Y1**
**1**									90.0
**2**									80.0
**3**									100.0
**4**									113.0
**5**									146.0
**6**									132.0
**7**									120.0
**8**									139.0

	Group C								
	XY24TL								
	**C:Y2**	**C:Y3**	**C:Y4**	**C:Y5**	**C:Y6**	**C:Y7**	**C:Y8**	**D:Y1**	**D:Y2**
**1**	98.0	105.0	88.0	94.0	102.0	94.0	102.0	93.0	100.0
**2**	99.0	93.0	89.0	84.0	103.0	84.0	103.0	84.0	109.0
**3**	134.0	116.0	121.0	104.0	140.0	104.0	140.0	123.0	110.0
**4**	98.0	131.0	88.0	118.0	102.0	118.0	102.0	129.0	110.0
**5**	132.0	168.0	119.0	152.0	138.0	152.0	138.0	150.0	164.0
**6**	119.0	152.0	107.0	138.0	124.0	138.0	124.0	110.0	166.0
**7**	138.0	139.0	125.0	125.0	144.0	125.0	144.0	159.0	148.0
**8**	119.0	160.0	107.0	145.0	124.0	145.0	124.0	109.0	130.0

	Group D								
	XY27FD								
	D:Y3	D:Y4	D:Y5	D:Y6	D:Y7	D:Y8	E:Y1	E:Y2	E:Y3
1	108.0	90.0	125.0	80.0	113.0	94.0	96.0	88.0	111.0
2	98.0	98.0	114.0	88.0	102.0	102.0	99.0	87.0	115.0
3	142.0	99.0	163.0	89.0	148.0	103.0	96.0	119.0	111.0
4	149.0	99.0	171.0	89.0	155.0	103.0	99.0	118.0	115.0
5	173.0	148.0	198.0	134.0	180.0	155.0	142.0	173.0	163.0
6	127.0	150.0	147.0	136.0	133.0	157.0	143.0	109.0	165.0
7	183.0	134.0	209.0	121.0	190.0	139.0	109.0	122.0	126.0
8	126.0	117.0	146.0	106.0	132.0	122.0	142.0	109.0	163.0

	Group E					Group			
	HIDEM 1					LGMD2D			
	**E:Y4**	**E:Y5**	**E:Y6**	**E:Y7**	**E:Y8**	**F:Y1**	**F:Y2**	**F:Y3**	**F:Y4**
**1**	79.0	129.0	70.0	116.0	82.0	89.0	90.0	104.0	81.0
**2**	78.0	133.0	69.0	120.0	81.0	88.0	108.0	102.0	97.0
**3**	107.0	129.0	96.0	116.0	112.0	99.0	90.0	115.0	81.0
**4**	106.0	133.0	95.0	120.0	111.0	108.0	120.0	125.0	108.0
**5**	157.0	188.0	142.0	170.0	163.0	183.0	133.0	210.0	120.0
**6**	98.0	189.0	88.0	171.0	102.0	123.0	112.0	142.0	101.0
**7**	110.0	146.0	99.0	132.0	115.0	157.0	134.0	180.0	121.0
**8**	98.0	188.0	88.0	170.0	102.0	150.0	119.0	173.0	107.0

Group F

LGMD2D Pt.3

	F:Y5	F:Y6	F:Y7	F:Y8
**1**	120.0	72.0	108.0	84.0
**2**	119.0	87.0	107.0	101.0
**3**	133.0	72.0	120.0	84.0
**4**	144.0	97.0	130.0	113.0
**5**	240.0	108.0	218.0	125.0
**6**	163.0	90.0	148.0	105.0
**7**	207.0	109.0	188.0	126.0
**8**	198.0	96.0	180.0	112.0
