# Supplementary material for: Human iPSC-derived mesoangioblasts, like their tissue-derived counterparts, suppress T cell proliferation through IDO- and PGE-2-dependent pathways
Source: F1000Res. 2013 Jan 25;2:24. [Version 1] doi: 10.12688/f1000research.2-24.v1 (PMC3968899; doi:10.12688/f1000research.2-24.v1)
Supplement: Raw data for Figure 2B: Change of surface marker expression of Mesoangioblasts/HIDEMs upon pro-inflammatory stimulation — HIDEMs and mesoangioblasts were stimulated with IFN-γ, TNF-α or IL-1β (20ng/ml) for 24h. Cells were trypsinized and washed, followed by surface staining for HLA-ABC, HLA-DR, CD40, PD-L1 or fluorochrome matched isotype controls and analysis by flow cytometry. Experiments were carried out in duplicates. n=4. Median fluorescence intensities of the markers were examined, and were shown as Mean ± SE. [file f1000research-2-1191-s0000.tgz › HLA_ABC_MFI.pdf]

Table format: **Grouped**		**Group A**							
		Data Set-A							
		**A:Y1**	**A:Y2**	**A:Y3**	**A:Y4**	**A:Y5**	**A:Y6**	**A:Y7**	**A:Y8**
**1**									
**2**									
**3**									
**4**									
**5**									
**6**									
**7**									
**8**									

	Group B								
	Data Set-B								
	B:Y1	B:Y2	B:Y3	B:Y4	B:Y5	B:Y6	B:Y7	B:Y8	C:Y1
1									1000.0
2									1800.0
3									1000.0
4									735.0
5									1436.0
6									1322.0
7									1000.0
8									1000.0

	Group C								
	XY24TL								
	C:Y2	C:Y3	C:Y4	C:Y5	C:Y6	C:Y7	C:Y8	D:Y1	D:Y2
1	980.0	1133.0	897.0	1283.0	821.0	1175.0	931.0	930.0	1100.0
2	1700.0	2037.0	1558.0	2305.0	1427.0	2113.0	1616.0	1900.0	1799.0
3	980.0	1133.0	897.0	1283.0	821.0	1175.0	931.0	736.0	1100.0
4	980.0	834.0	897.0	945.0	821.0	865.0	931.0	930.0	893.0
5	1782.0	1626.0	1633.0	1840.0	1496.0	1686.0	1694.0	1837.0	1648.0
6	980.0	1497.0	897.0	1694.0	821.0	1553.0	931.0	900.0	1660.0
7	980.0	1133.0	897.0	1283.0	821.0	1175.0	931.0	930.0	1100.0
8	1193.0	1133.0	1092.0	1283.0	1000.0	1175.0	1133.0	930.0	1100.0

	Group D XY27FD								
	D:Y3	D:Y4	D:Y5	D:Y6	D:Y7	D:Y8	E:Y1	E:Y2	E:Y3
1	1054.0	1007.0	1194.0	922.0	1093.0	1045.0	960.0	980.0	1088.0
2	2150.0	1648.0	2433.0	1510.0	2230.0	1710.0	1798.0	1965.0	2035.0
3	835.0	1007.0	946.0	922.0	866.0	1045.0	960.0	872.0	1088.0
4	1054.0	817.0	1194.0	748.0	1093.0	848.0	789.0	980.0	895.0
5	2079.0	1510.0	2352.0	1383.0	2156.0	1566.0	1927.0	1739.0	2181.0
6	1020.0	1521.0	1156.0	1393.0	1058.0	1577.0	1431.0	899.0	1620.0
7	1054.0	1007.0	1194.0	922.0	1093.0	1045.0	960.0	725.0	1088.0
8	1054.0	1007.0	1194.0	922.0	1093.0	1045.0	1421.0	980.0	1609.0

	Group E					Group			
	HIDEM 1					LGMD2D HIDEM			
	E:Y4	E:Y5	E:Y6	E:Y7	E:Y8	F:Y1	F:Y2	F:Y3	F:Y4
1	897.0	1232.0	821.0	1128.0	931.0	990.0	900.0	1122.0	824.0
2	1801.0	2302.0	1650.0	2110.0	1868.0	1888.0	1682.0	2136.0	1541.0
3	798.0	1232.0	730.0	1128.0	828.0	990.0	900.0	1122.0	824.0
4	897.0	1014.0	821.0	928.0	931.0	788.0	900.0	893.0	824.0
5	1593.0	2467.0	1460.0	2261.0	1653.0	1836.0	1638.0	2078.0	1501.0
6	823.0	1834.0	753.0	1680.0	854.0	990.0	1123.0	1122.0	1028.0
7	663.0	1232.0	606.0	1128.0	688.0	990.0	900.0	1122.0	824.0
8	897.0	1821.0	821.0	1669.0	931.0	1200.0	900.0	1359.0	824.0

Group F<br>LGMD2D Pt.3<br>HIDEMs	F:Y5	F:Y6	F:Y7	F:Y8
**1**	1271.0	754.0	1164.0	855.0
**2**	2417.0	1412.0	2216.0	1598.0
**3**	1271.0	754.0	1164.0	855.0
**4**	1013.0	754.0	927.0	855.0
**5**	2351.0	1375.0	2155.0	1557.0
**6**	1271.0	941.0	1164.0	1067.0
**7**	1271.0	754.0	1164.0	855.0
**8**	1539.0	754.0	1410.0	855.0
