# Supplementary material for: Human iPSC-derived mesoangioblasts, like their tissue-derived counterparts, suppress T cell proliferation through IDO- and PGE-2-dependent pathways
Source: F1000Res. 2013 Jan 25;2:24. [Version 1] doi: 10.12688/f1000research.2-24.v1 (PMC3968899; doi:10.12688/f1000research.2-24.v1)
Supplement: Raw data for Figure 2B: Change of surface marker expression of Mesoangioblasts/HIDEMs upon pro-inflammatory stimulation — HIDEMs and mesoangioblasts were stimulated with IFN-γ, TNF-α or IL-1β (20ng/ml) for 24h. Cells were trypsinized and washed, followed by surface staining for HLA-ABC, HLA-DR, CD40, PD-L1 or fluorochrome matched isotype controls and analysis by flow cytometry. Experiments were carried out in duplicates. n=4. Median fluorescence intensities of the markers were examined, and were shown as Mean ± SE. [file f1000research-2-1191-s0000.tgz › HLA_DR_MFI.pdf]

Table format: **Grouped**		**Group A**							
		Data Set-A							
		**A:Y1**	**A:Y2**	**A:Y3**	**A:Y4**	**A:Y5**	**A:Y6**	**A:Y7**	**A:Y8**
**1**									
**2**									
**3**									
**4**									
**5**									
**6**									
**7**									
**8**									

	Group B								
	Data Set-B								
	**B:Y1**	**B:Y2**	**B:Y3**	**B:Y4**	**B:Y5**	**B:Y6**	**B:Y7**	**B:Y8**	**C:Y1**
**1**									200.0
**2**									380.0
**3**									166.0
**4**									148.0
**5**									379.0
**6**									200.0
**7**									200.0
**8**									200.0

	Group C								
	XY24TL								
	**C:Y2**	**C:Y3**	**C:Y4**	**C:Y5**	**C:Y6**	**C:Y7**	**C:Y8**	**D:Y1**	**D:Y2**
**1**	189.0	229.0	171.0	262.0	155.0	238.0	178.0	210.0	177.0
**2**	270.0	432.0	246.0	492.0	223.0	449.0	255.0	366.0	324.0
**3**	189.0	191.0	171.0	218.0	155.0	198.0	178.0	210.0	177.0
**4**	155.0	170.0	140.0	195.0	127.0	177.0	146.0	185.0	179.0
**5**	300.0	431.0	273.0	490.0	249.0	448.0	284.0	288.0	290.0
**6**	267.0	229.0	243.0	262.0	221.0	238.0	253.0	210.0	277.0
**7**	222.0	229.0	202.0	262.0	183.0	238.0	210.0	210.0	256.0
**8**	218.0	229.0	198.0	262.0	180.0	238.0	206.0	270.0	234.0

	Group D								
	XY27FD								
	D:Y3	D:Y4	D:Y5	D:Y6	D:Y7	D:Y8	E:Y1	E:Y2	E:Y3
1	240.0	160.0	275.0	145.0	250.0	167.0	190.0	209.0	218.0
2	417.0	295.0	474.0	269.0	433.0	307.0	320.0	294.0	365.0
3	240.0	160.0	275.0	145.0	250.0	167.0	190.0	170.0	218.0
4	212.0	162.0	243.0	147.0	221.0	169.0	152.0	160.0	175.0
5	328.0	264.0	374.0	240.0	341.0	274.0	302.0	279.0	344.0
6	240.0	252.0	275.0	229.0	250.0	262.0	190.0	209.0	218.0
7	240.0	233.0	275.0	212.0	250.0	242.0	190.0	209.0	218.0
8	308.0	213.0	351.0	193.0	320.0	221.0	240.0	229.0	274.0

	Group E					Group			
	HIDEM 1					LGMD2D			
	E:Y4	E:Y5	E:Y6	E:Y7	E:Y8	F:Y1	F:Y2	F:Y3	F:Y4
1	190.0	249.0	172.0	226.0	197.0	197.0	206.0	226.0	187.0
2	268.0	415.0	244.0	379.0	278.0	288.0	290.0	328.0	264.0
3	154.0	249.0	139.0	226.0	160.0	197.0	206.0	226.0	187.0
4	145.0	200.0	131.0	182.0	151.0	170.0	189.0	195.0	171.0
5	254.0	392.0	231.0	358.0	264.0	340.0	287.0	387.0	261.0
6	190.0	249.0	172.0	226.0	197.0	297.0	206.0	339.0	187.0
7	190.0	249.0	172.0	226.0	197.0	197.0	206.0	226.0	187.0
8	208.0	313.0	189.0	285.0	216.0	255.0	226.0	291.0	205.0

Group F				
LGMD2D Pt.3				
	F:Y5	F:Y6	F:Y7	F:Y8
1	258.0	170.0	235.0	195.0
2	374.0	240.0	341.0	274.0
3	258.0	170.0	235.0	195.0
4	223.0	155.0	203.0	178.0
5	441.0	238.0	402.0	272.0
6	386.0	170.0	352.0	195.0
7	258.0	170.0	235.0	195.0
8	332.0	186.0	303.0	214.0
