# Supplementary material for: Human iPSC-derived mesoangioblasts, like their tissue-derived counterparts, suppress T cell proliferation through IDO- and PGE-2-dependent pathways
Source: F1000Res. 2013 Jan 25;2:24. [Version 1] doi: 10.12688/f1000research.2-24.v1 (PMC3968899; doi:10.12688/f1000research.2-24.v1)
Supplement: Raw data for Figure 2B: Change of surface marker expression of Mesoangioblasts/HIDEMs upon pro-inflammatory stimulation — HIDEMs and mesoangioblasts were stimulated with IFN-γ, TNF-α or IL-1β (20ng/ml) for 24h. Cells were trypsinized and washed, followed by surface staining for HLA-ABC, HLA-DR, CD40, PD-L1 or fluorochrome matched isotype controls and analysis by flow cytometry. Experiments were carried out in duplicates. n=4. Median fluorescence intensities of the markers were examined, and were shown as Mean ± SE. [file f1000research-2-1191-s0000.tgz › PD_L1_MFI.pdf]

Table format: **Grouped**		Group A							
		Data Set-A							
		**A:Y1**	**A:Y2**	**A:Y3**	**A:Y4**	**A:Y5**	**A:Y6**	**A:Y7**	**A:Y8**
**1**									
**2**									
**3**									
**4**									
**5**									
**6**									
**7**									
**8**									

	Group B								
	Data Set-B								
	**B:Y1**	**B:Y2**	**B:Y3**	**B:Y4**	**B:Y5**	**B:Y6**	**B:Y7**	**B:Y8**	**C:Y1**
**1**									100.0
**2**									380.0
**3**									109.0
**4**									90.0
**5**									379.0
**6**									309.0
**7**									99.0
**8**									345.0

	Group C								
	XY24TL								
	C:Y2	C:Y3	C:Y4	C:Y5	C:Y6	C:Y7	C:Y8	D:Y1	D:Y2
1	98.0	116.0	88.0	134.0	79.0	121.0	92.0	110.0	98.0
2	309.0	432.0	281.0	492.0	256.0	449.0	293.0	366.0	309.0
3	89.0	126.0	80.0	146.0	71.0	132.0	83.0	110.0	89.0
4	97.0	105.0	87.0	121.0	78.0	109.0	91.0	85.0	97.0
5	455.0	431.0	415.0	490.0	379.0	448.0	431.0	288.0	455.0
6	311.0	352.0	283.0	401.0	258.0	366.0	294.0	288.0	311.0
7	89.0	115.0	80.0	133.0	71.0	120.0	83.0	110.0	89.0
8	281.0	393.0	256.0	447.0	233.0	408.0	266.0	365.0	281.0

	Group D								
	XY27FD								
	D:Y3	D:Y4	D:Y5	D:Y6	D:Y7	D:Y8	E:Y1	E:Y2	E:Y3
1	127.0	88.0	147.0	79.0	133.0	92.0	110.0	98.0	127.0
2	417.0	281.0	474.0	256.0	433.0	293.0	366.0	309.0	417.0
3	127.0	80.0	147.0	71.0	133.0	83.0	110.0	89.0	127.0
4	99.0	87.0	115.0	78.0	103.0	91.0	85.0	97.0	99.0
5	328.0	415.0	374.0	379.0	341.0	431.0	288.0	455.0	328.0
6	328.0	283.0	374.0	258.0	341.0	294.0	288.0	311.0	328.0
7	127.0	80.0	147.0	71.0	133.0	83.0	110.0	89.0	127.0
8	415.0	256.0	472.0	233.0	431.0	266.0	365.0	281.0	415.0

	Group E					Group			
	HIDEM 1					LGMD2D			
	E:Y4	E:Y5	E:Y6	E:Y7	E:Y8	F:Y1	F:Y2	F:Y3	F:Y4
1	88.0	147.0	79.0	133.0	92.0	110.0	109.0	127.0	98.0
2	281.0	474.0	256.0	433.0	293.0	366.0	294.0	417.0	268.0
3	80.0	147.0	71.0	133.0	83.0	110.0	90.0	127.0	81.0
4	87.0	115.0	78.0	103.0	91.0	85.0	90.0	99.0	81.0
5	415.0	374.0	379.0	341.0	431.0	288.0	330.0	328.0	301.0
6	283.0	374.0	258.0	341.0	294.0	288.0	324.0	328.0	295.0
7	80.0	147.0	71.0	133.0	83.0	110.0	109.0	127.0	98.0
8	256.0	472.0	233.0	431.0	266.0	365.0	220.0	415.0	200.0

Group F				
LGMD2D Pt.3				
	F:Y5	F:Y6	F:Y7	F:Y8
**1**	147.0	88.0	133.0	102.0
**2**	474.0	244.0	433.0	278.0
**3**	147.0	72.0	133.0	84.0
**4**	115.0	72.0	103.0	84.0
**5**	374.0	274.0	341.0	313.0
**6**	374.0	269.0	341.0	307.0
**7**	147.0	88.0	133.0	102.0
**8**	472.0	181.0	431.0	208.0
