# Supplementary figures and images for: Human iPSC-derived mesoangioblasts, like their tissue-derived counterparts, suppress T cell proliferation through IDO- and PGE-2-dependent pathways
Source: F1000Res. 2013 Jan 25;2:24. [Version 1] doi: 10.12688/f1000research.2-24.v1 (PMC3968899; doi:10.12688/f1000research.2-24.v1)

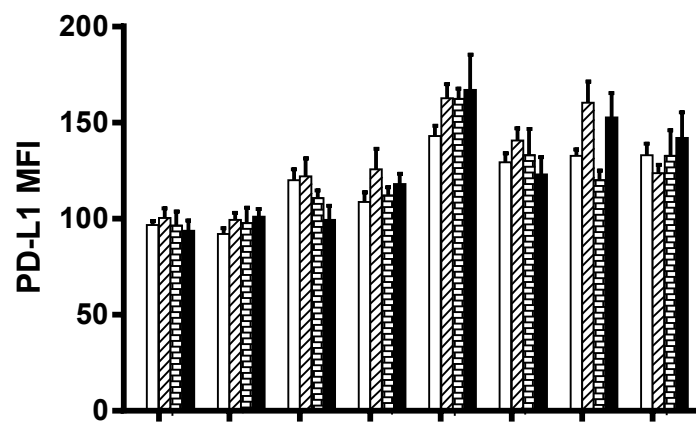

Supplement: Raw data for Figure 2B: Change of surface marker expression of Mesoangioblasts/HIDEMs upon pro-inflammatory stimulation — HIDEMs and mesoangioblasts were stimulated with IFN-γ, TNF-α or IL-1β (20ng/ml) for 24h. Cells were trypsinized and washed, followed by surface staining for HLA-ABC, HLA-DR, CD40, PD-L1 or fluorochrome matched isotype controls and analysis by flow cytometry. Experiments were carried out in duplicates. n=4. Median fluorescence intensities of the markers were examined, and were shown as Mean ± SE. [file f1000research-2-1191-s0000.tgz › CD40_MFI_graph.pdf]

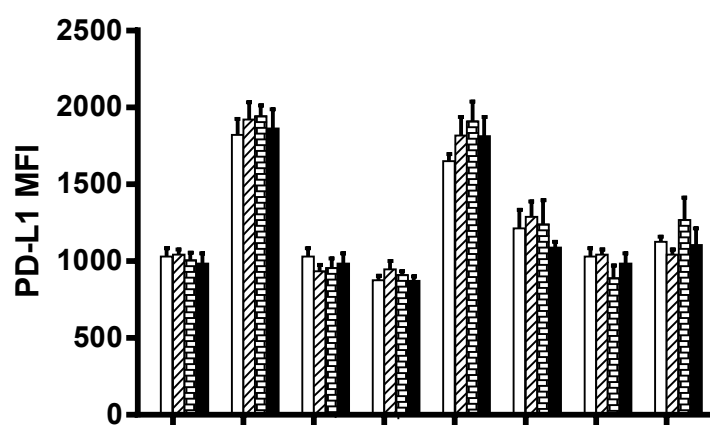

Supplement: Raw data for Figure 2B: Change of surface marker expression of Mesoangioblasts/HIDEMs upon pro-inflammatory stimulation — HIDEMs and mesoangioblasts were stimulated with IFN-γ, TNF-α or IL-1β (20ng/ml) for 24h. Cells were trypsinized and washed, followed by surface staining for HLA-ABC, HLA-DR, CD40, PD-L1 or fluorochrome matched isotype controls and analysis by flow cytometry. Experiments were carried out in duplicates. n=4. Median fluorescence intensities of the markers were examined, and were shown as Mean ± SE. [file f1000research-2-1191-s0000.tgz › HLA_ABC_MFI_graph.pdf]

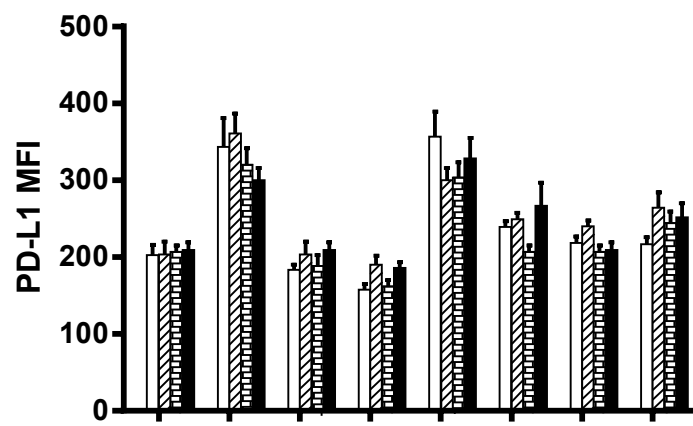

Supplement: Raw data for Figure 2B: Change of surface marker expression of Mesoangioblasts/HIDEMs upon pro-inflammatory stimulation — HIDEMs and mesoangioblasts were stimulated with IFN-γ, TNF-α or IL-1β (20ng/ml) for 24h. Cells were trypsinized and washed, followed by surface staining for HLA-ABC, HLA-DR, CD40, PD-L1 or fluorochrome matched isotype controls and analysis by flow cytometry. Experiments were carried out in duplicates. n=4. Median fluorescence intensities of the markers were examined, and were shown as Mean ± SE. [file f1000research-2-1191-s0000.tgz › HLA_DR_MFI_graph.pdf]

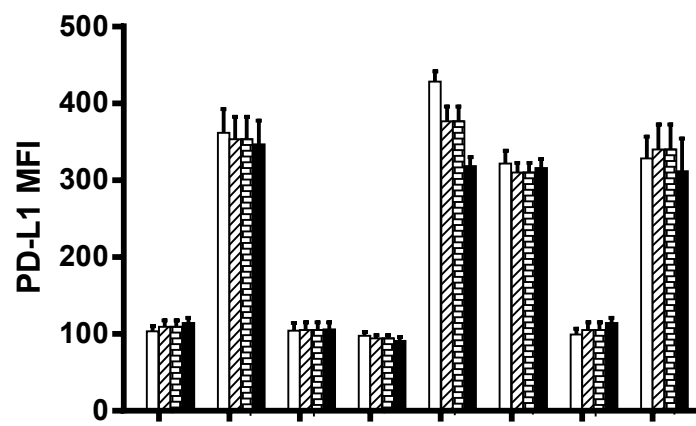

Supplement: Raw data for Figure 2B: Change of surface marker expression of Mesoangioblasts/HIDEMs upon pro-inflammatory stimulation — HIDEMs and mesoangioblasts were stimulated with IFN-γ, TNF-α or IL-1β (20ng/ml) for 24h. Cells were trypsinized and washed, followed by surface staining for HLA-ABC, HLA-DR, CD40, PD-L1 or fluorochrome matched isotype controls and analysis by flow cytometry. Experiments were carried out in duplicates. n=4. Median fluorescence intensities of the markers were examined, and were shown as Mean ± SE. [file f1000research-2-1191-s0000.tgz › PD_L1_MFI_graph.pdf]

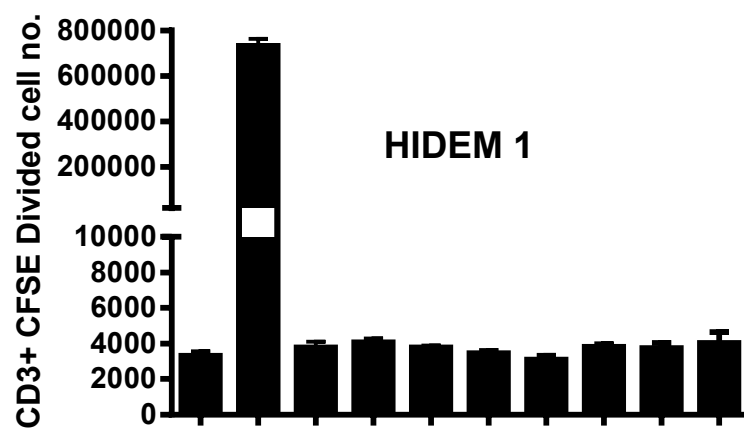

Supplement: Raw data for Figure 2C: HIDEMs and mesoangioblasts fail to induce T cell proliferation in vitro — CFSE labelled PBMCs were stimulated with anti CD3/CD28 beads (PBMC+B) as a positive control. HIDEMs and mesoangioblasts were stimulated with IFN-γ, TNF-α or IL-1β (20ng/ml) for 24h. Non-stimulated or cytokine stimulated HIDEMs/mesoangioblasts (ratio 1:4) were then co-cultured with PBMC for 6 days. CD3+ CFSE labelled 7AAD- cells were enumerated using flow cytometry and counting beads. Experiments were carried out in duplicates. n=4. [file f1000research-2-1191-s0001.tgz › Immunogenicity_HIDEM_1_graph.pdf]

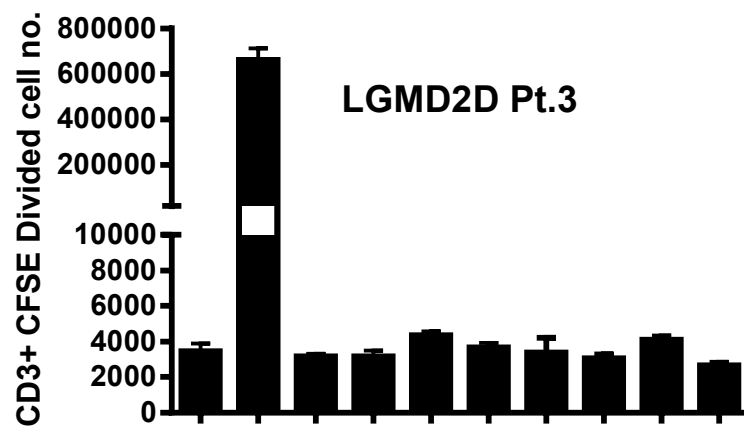

Supplement: Raw data for Figure 2C: HIDEMs and mesoangioblasts fail to induce T cell proliferation in vitro — CFSE labelled PBMCs were stimulated with anti CD3/CD28 beads (PBMC+B) as a positive control. HIDEMs and mesoangioblasts were stimulated with IFN-γ, TNF-α or IL-1β (20ng/ml) for 24h. Non-stimulated or cytokine stimulated HIDEMs/mesoangioblasts (ratio 1:4) were then co-cultured with PBMC for 6 days. CD3+ CFSE labelled 7AAD- cells were enumerated using flow cytometry and counting beads. Experiments were carried out in duplicates. n=4. [file f1000research-2-1191-s0001.tgz › Immunogenicity_LGMD2D_Pt3_graph.pdf]

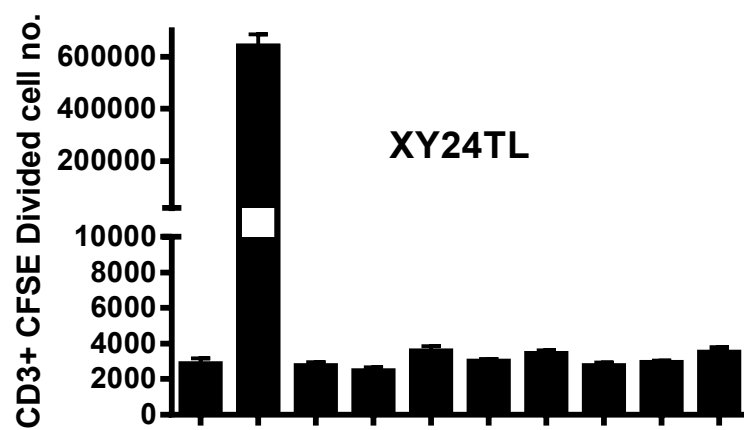

Supplement: Raw data for Figure 2C: HIDEMs and mesoangioblasts fail to induce T cell proliferation in vitro — CFSE labelled PBMCs were stimulated with anti CD3/CD28 beads (PBMC+B) as a positive control. HIDEMs and mesoangioblasts were stimulated with IFN-γ, TNF-α or IL-1β (20ng/ml) for 24h. Non-stimulated or cytokine stimulated HIDEMs/mesoangioblasts (ratio 1:4) were then co-cultured with PBMC for 6 days. CD3+ CFSE labelled 7AAD- cells were enumerated using flow cytometry and counting beads. Experiments were carried out in duplicates. n=4. [file f1000research-2-1191-s0001.tgz › Immunogenicity_XY24TL_graph.pdf]

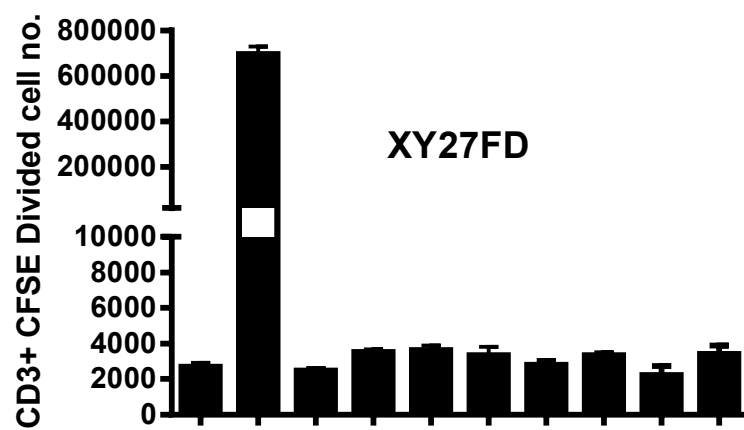

Supplement: Raw data for Figure 2C: HIDEMs and mesoangioblasts fail to induce T cell proliferation in vitro — CFSE labelled PBMCs were stimulated with anti CD3/CD28 beads (PBMC+B) as a positive control. HIDEMs and mesoangioblasts were stimulated with IFN-γ, TNF-α or IL-1β (20ng/ml) for 24h. Non-stimulated or cytokine stimulated HIDEMs/mesoangioblasts (ratio 1:4) were then co-cultured with PBMC for 6 days. CD3+ CFSE labelled 7AAD- cells were enumerated using flow cytometry and counting beads. Experiments were carried out in duplicates. n=4. [file f1000research-2-1191-s0001.tgz › Immunogenicity_XY27FD_graph.pdf]

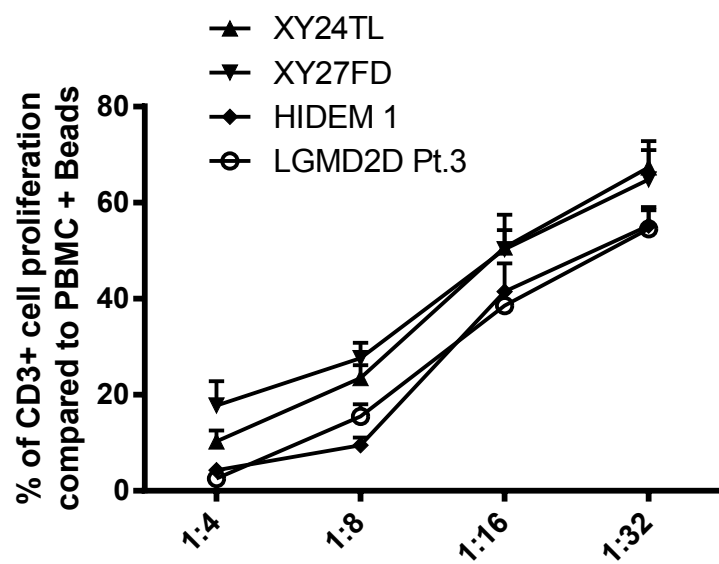

Supplement: Raw data for Figure 3B: Mesoangioblasts and HIDEMs suppress T cell proliferation in a dose dependent manner — CFSE labelled PBMCs (5 x 10^4^/well) were stimulated with anti CD3/CD28 beads (1 x 10^4^/well) (P+B) in the presence or absence of HIDEMs/mesoangioblasts at decreasing ratios (HIDEM/mesoangioblast:PBMC). On day 6 cells were harvested and stained with anti-CD3 antibody and 7AAD, and analysed by flow cytometry. CFSE dilution was analysed on gated CD3+ 7AAD- cells. The percentage of CD3+CFSE dividing cells was calculated for each group and compared to the positive control (P+B), followed by plotting against HIDEM/mesoangioblast:PBMC ratios. Experiments were carried out in duplicates. n=2 [file f1000research-2-1191-s0002.tgz › HIDEMs_graph.pdf]

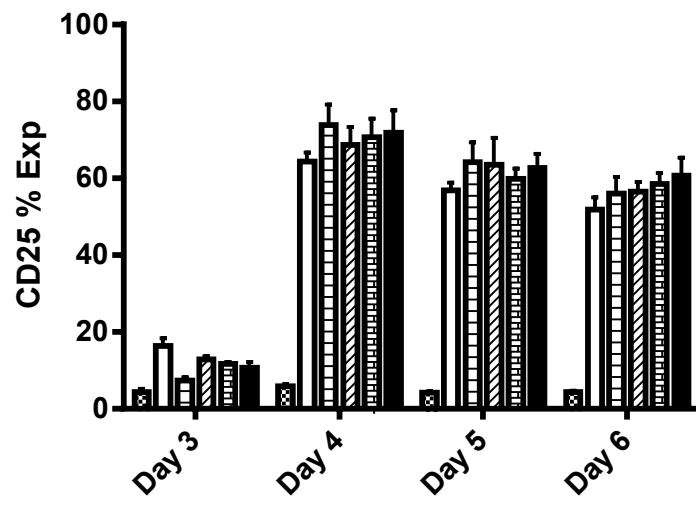

Supplement: Raw data for Figure 3C: Mesoangioblasts and HIDEMs do not interfer with T cell activation — CFSE labelled PBMCs (5 x 10^4^/well) were stimulated with anti CD3/CD28 beads (1 x 10^4^/well) (P+B) in the presence or absence of HIDEMs/mesoangioblasts at HIDEM/mesoangioblast:PBMC = 1:4 ratio. Cells were harvested on day 3, 4, 5 or 6 and analysed for CFSE dilution and expression of CD25 and CD69. The number of CD3+7AAD- cells expressing CD25 or CD69 using counting beads and the % of CD25+ and CD69+ cells were calculated from the data. Experiments were carried out in duplicates. n=2. [file f1000research-2-1191-s0003.tgz › __of_CD25_expressing_T_cells_graph.pdf]

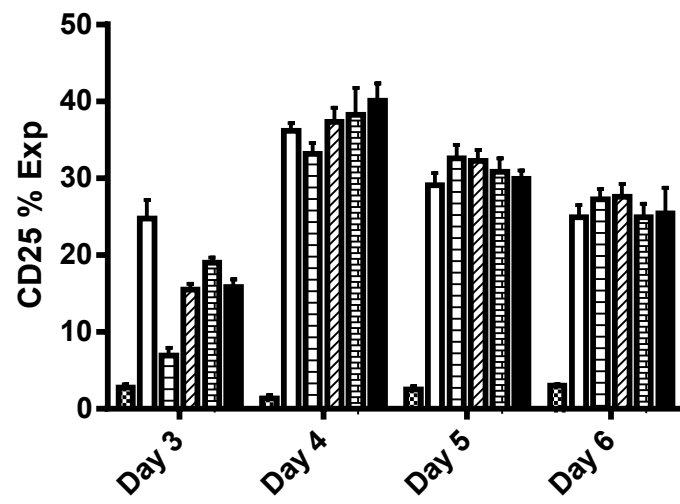

Supplement: Raw data for Figure 3C: Mesoangioblasts and HIDEMs do not interfer with T cell activation — CFSE labelled PBMCs (5 x 10^4^/well) were stimulated with anti CD3/CD28 beads (1 x 10^4^/well) (P+B) in the presence or absence of HIDEMs/mesoangioblasts at HIDEM/mesoangioblast:PBMC = 1:4 ratio. Cells were harvested on day 3, 4, 5 or 6 and analysed for CFSE dilution and expression of CD25 and CD69. The number of CD3+7AAD- cells expressing CD25 or CD69 using counting beads and the % of CD25+ and CD69+ cells were calculated from the data. Experiments were carried out in duplicates. n=2. [file f1000research-2-1191-s0003.tgz › __of_CD69_expressing_T_cells_graph.pdf]

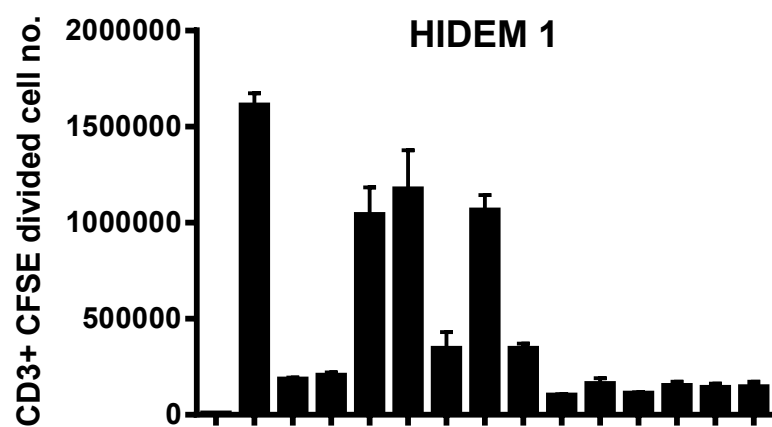

Supplement: Raw data for Figure 4A: Neutralising antibodies against IFN-γ and TNF-α reduce the immunosuppressive capacity of Mesoangioblasts/HIDEMs — CFSE labelled PBMCs were stimulated with anti-CD3/CD28 beads in the presence of HIDEMs/mesoangioblasts (1:4) and neutralising antibodies against IFN-γ and TNF-α or irrelevant isotype control antibody (0.5, 1.0 and 2.0 µg/ml) or recombinant IL-1RA (0.5, 1.0 and 2.0 µg/ml). Cells were harvested on day 6 and stained with anti-CD3 and 7AAD. After gating on CD3+7AAD- the number of CFSE diluting cells were enumerated using counting beads. Experiments were carried out in duplicates. n=4. [file f1000research-2-1191-s0004.tgz › HIDEM_1_graph.pdf]

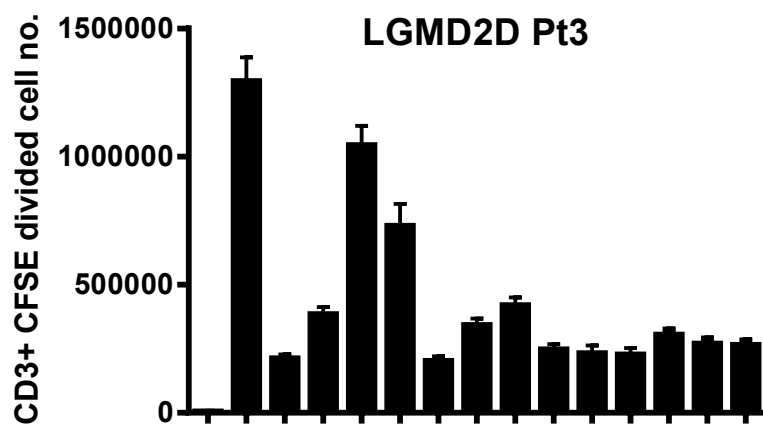

Supplement: Raw data for Figure 4A: Neutralising antibodies against IFN-γ and TNF-α reduce the immunosuppressive capacity of Mesoangioblasts/HIDEMs — CFSE labelled PBMCs were stimulated with anti-CD3/CD28 beads in the presence of HIDEMs/mesoangioblasts (1:4) and neutralising antibodies against IFN-γ and TNF-α or irrelevant isotype control antibody (0.5, 1.0 and 2.0 µg/ml) or recombinant IL-1RA (0.5, 1.0 and 2.0 µg/ml). Cells were harvested on day 6 and stained with anti-CD3 and 7AAD. After gating on CD3+7AAD- the number of CFSE diluting cells were enumerated using counting beads. Experiments were carried out in duplicates. n=4. [file f1000research-2-1191-s0004.tgz › LGMD2D_Pt3_graph.pdf]

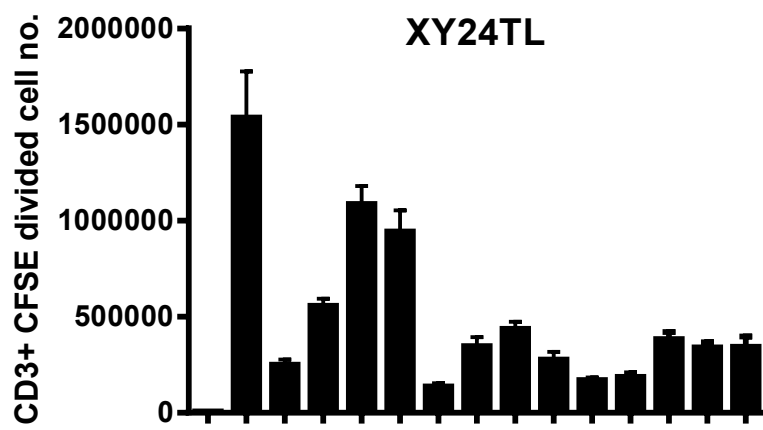

Supplement: Raw data for Figure 4A: Neutralising antibodies against IFN-γ and TNF-α reduce the immunosuppressive capacity of Mesoangioblasts/HIDEMs — CFSE labelled PBMCs were stimulated with anti-CD3/CD28 beads in the presence of HIDEMs/mesoangioblasts (1:4) and neutralising antibodies against IFN-γ and TNF-α or irrelevant isotype control antibody (0.5, 1.0 and 2.0 µg/ml) or recombinant IL-1RA (0.5, 1.0 and 2.0 µg/ml). Cells were harvested on day 6 and stained with anti-CD3 and 7AAD. After gating on CD3+7AAD- the number of CFSE diluting cells were enumerated using counting beads. Experiments were carried out in duplicates. n=4. [file f1000research-2-1191-s0004.tgz › XY24TL_graph.pdf]

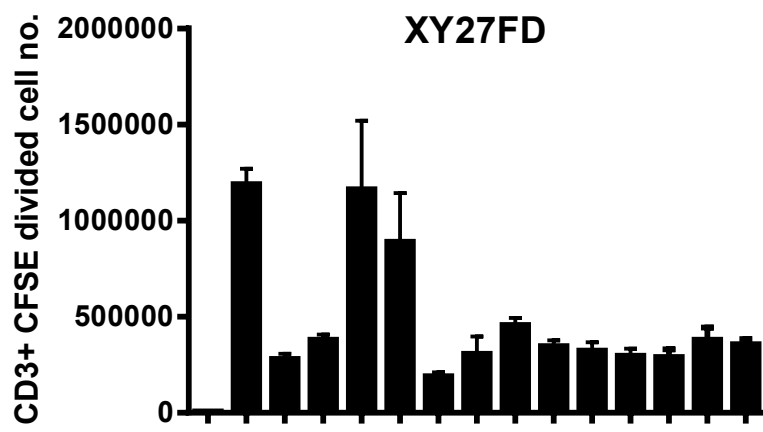

Supplement: Raw data for Figure 4A: Neutralising antibodies against IFN-γ and TNF-α reduce the immunosuppressive capacity of Mesoangioblasts/HIDEMs — CFSE labelled PBMCs were stimulated with anti-CD3/CD28 beads in the presence of HIDEMs/mesoangioblasts (1:4) and neutralising antibodies against IFN-γ and TNF-α or irrelevant isotype control antibody (0.5, 1.0 and 2.0 µg/ml) or recombinant IL-1RA (0.5, 1.0 and 2.0 µg/ml). Cells were harvested on day 6 and stained with anti-CD3 and 7AAD. After gating on CD3+7AAD- the number of CFSE diluting cells were enumerated using counting beads. Experiments were carried out in duplicates. n=4. [file f1000research-2-1191-s0004.tgz › XY27FD_graph.pdf]

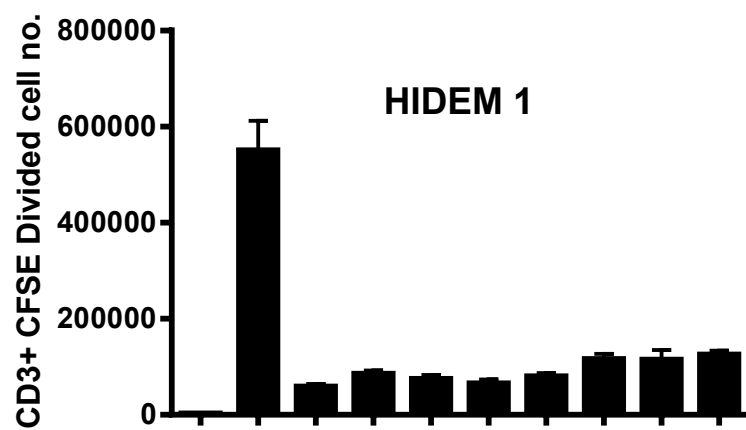

Supplement: Raw data for Figure 4B: Pre-stimulation with IFN-γ, TNF-α and IL-1β does not enhance the immunosuppressive effect of Mesoangioblasts/HIDEMs — HIDEMs/mesoangioblasts were left untreated or were stimulated with IFN-γ, TNF-α or IL-1β (20ng/ml) for 24h before setting up co-cultures with CFSE labelled PBMC and anti CD3/CD28 beads. After 6 days cells were harvested and surface stained for CD3 and 7AAD before analysis of CFSE dilution. CD3+CFSE diluted cell numbers were calculated using counting beads as before. Experiments were carried out in duplicates. n=4. [file f1000research-2-1191-s0005.tgz › HIDEM_1_graph.pdf]

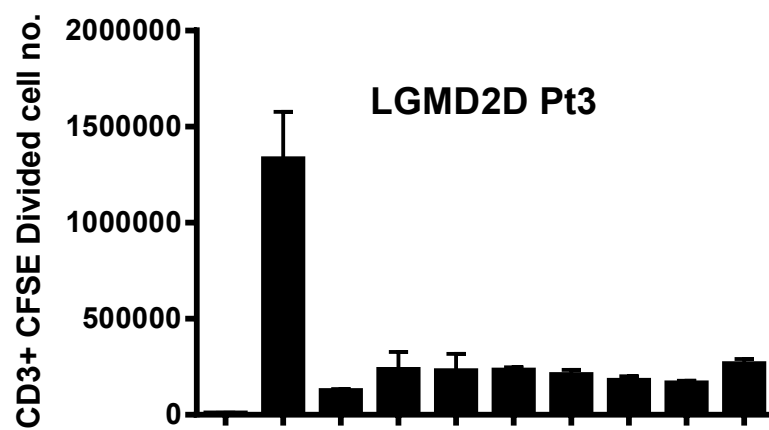

Supplement: Raw data for Figure 4B: Pre-stimulation with IFN-γ, TNF-α and IL-1β does not enhance the immunosuppressive effect of Mesoangioblasts/HIDEMs — HIDEMs/mesoangioblasts were left untreated or were stimulated with IFN-γ, TNF-α or IL-1β (20ng/ml) for 24h before setting up co-cultures with CFSE labelled PBMC and anti CD3/CD28 beads. After 6 days cells were harvested and surface stained for CD3 and 7AAD before analysis of CFSE dilution. CD3+CFSE diluted cell numbers were calculated using counting beads as before. Experiments were carried out in duplicates. n=4. [file f1000research-2-1191-s0005.tgz › LGMD2D_Pt3_graph.pdf]

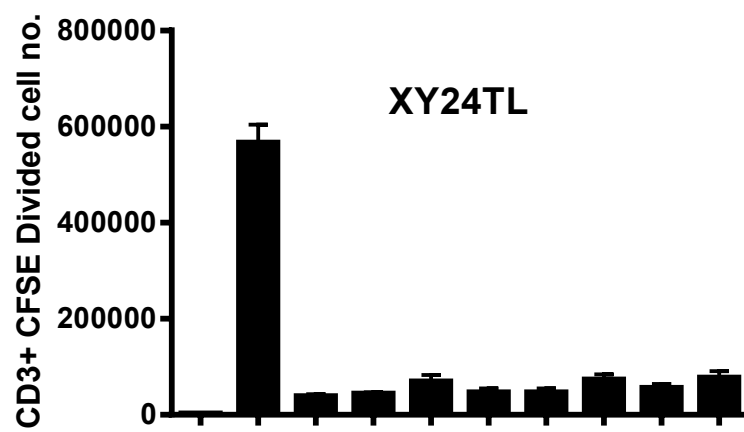

Supplement: Raw data for Figure 4B: Pre-stimulation with IFN-γ, TNF-α and IL-1β does not enhance the immunosuppressive effect of Mesoangioblasts/HIDEMs — HIDEMs/mesoangioblasts were left untreated or were stimulated with IFN-γ, TNF-α or IL-1β (20ng/ml) for 24h before setting up co-cultures with CFSE labelled PBMC and anti CD3/CD28 beads. After 6 days cells were harvested and surface stained for CD3 and 7AAD before analysis of CFSE dilution. CD3+CFSE diluted cell numbers were calculated using counting beads as before. Experiments were carried out in duplicates. n=4. [file f1000research-2-1191-s0005.tgz › XY24TL_graph.pdf]

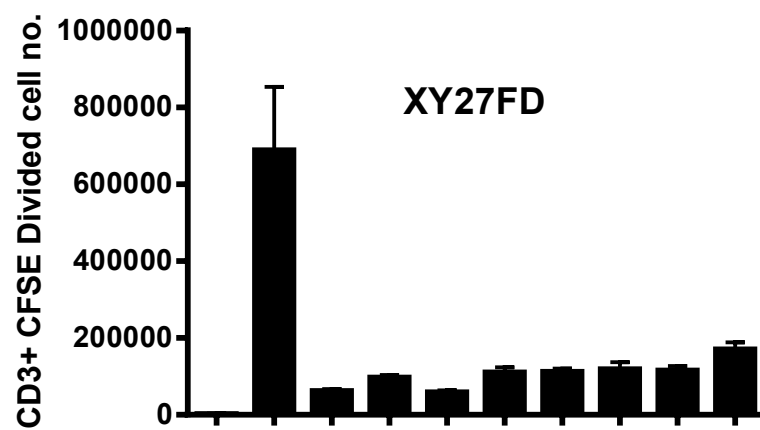

Supplement: Raw data for Figure 4B: Pre-stimulation with IFN-γ, TNF-α and IL-1β does not enhance the immunosuppressive effect of Mesoangioblasts/HIDEMs — HIDEMs/mesoangioblasts were left untreated or were stimulated with IFN-γ, TNF-α or IL-1β (20ng/ml) for 24h before setting up co-cultures with CFSE labelled PBMC and anti CD3/CD28 beads. After 6 days cells were harvested and surface stained for CD3 and 7AAD before analysis of CFSE dilution. CD3+CFSE diluted cell numbers were calculated using counting beads as before. Experiments were carried out in duplicates. n=4. [file f1000research-2-1191-s0005.tgz › XY27FD_graph.pdf]

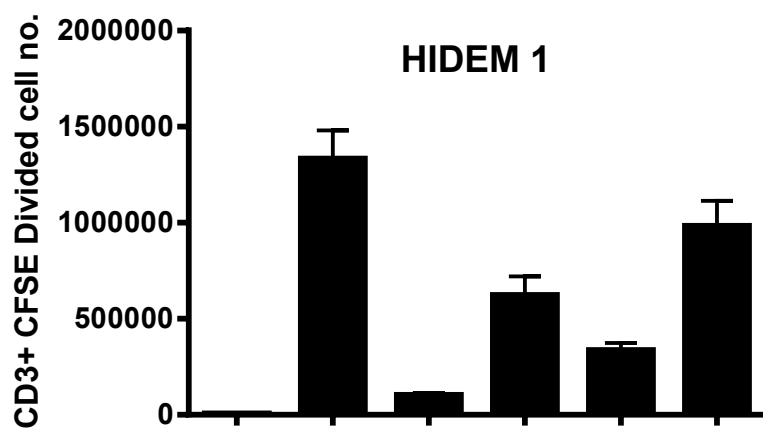

Supplement: Raw data for Figure 5: The presence of IDO and PGE-2 inhibitors reduce the suppression of T cell proliferation by Mesoangioblasts/HIDEMs — CFSE labelled PBMCs were stimulated with anti CD3/CD28 beads as before in the presence of HIDEMs/mesoangioblasts and inhibitors of IDO and Cox-2, (1-Methyl-L-trypyophan (1MT) (0.5mM) and NS-398 (1.0 uM) respectively, or both. On day 6 cells were harvested and stained with anti-CD3 and 7AAD. Cells were gated on live CD3+ populations and analysed for CFSE dilution and the numbers of cells undergoing CFSE dilution were enumerated using counting beads. Experiments were carried out in duplicates. n=4. [file f1000research-2-1191-s0006.tgz › HIDEM_1_graph.pdf]

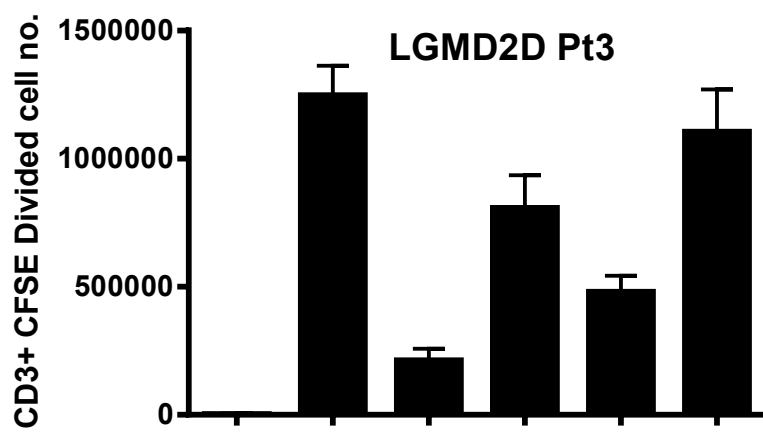

Supplement: Raw data for Figure 5: The presence of IDO and PGE-2 inhibitors reduce the suppression of T cell proliferation by Mesoangioblasts/HIDEMs — CFSE labelled PBMCs were stimulated with anti CD3/CD28 beads as before in the presence of HIDEMs/mesoangioblasts and inhibitors of IDO and Cox-2, (1-Methyl-L-trypyophan (1MT) (0.5mM) and NS-398 (1.0 uM) respectively, or both. On day 6 cells were harvested and stained with anti-CD3 and 7AAD. Cells were gated on live CD3+ populations and analysed for CFSE dilution and the numbers of cells undergoing CFSE dilution were enumerated using counting beads. Experiments were carried out in duplicates. n=4. [file f1000research-2-1191-s0006.tgz › LGMD2D_Pt3_graph.pdf]

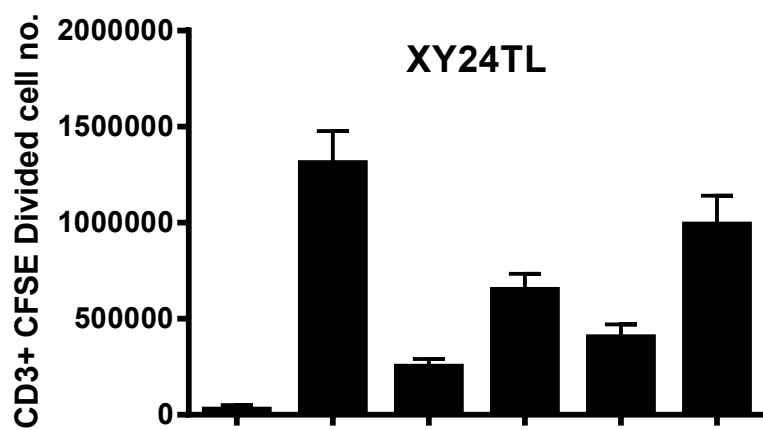

Supplement: Raw data for Figure 5: The presence of IDO and PGE-2 inhibitors reduce the suppression of T cell proliferation by Mesoangioblasts/HIDEMs — CFSE labelled PBMCs were stimulated with anti CD3/CD28 beads as before in the presence of HIDEMs/mesoangioblasts and inhibitors of IDO and Cox-2, (1-Methyl-L-trypyophan (1MT) (0.5mM) and NS-398 (1.0 uM) respectively, or both. On day 6 cells were harvested and stained with anti-CD3 and 7AAD. Cells were gated on live CD3+ populations and analysed for CFSE dilution and the numbers of cells undergoing CFSE dilution were enumerated using counting beads. Experiments were carried out in duplicates. n=4. [file f1000research-2-1191-s0006.tgz › XY24TL_graph.pdf]

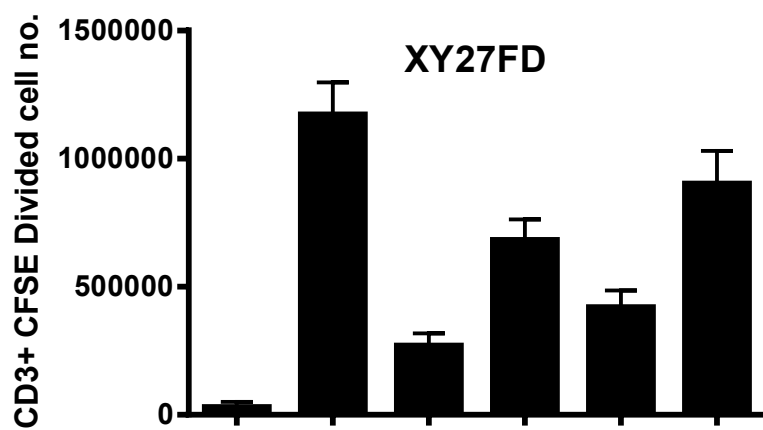

Supplement: Raw data for Figure 5: The presence of IDO and PGE-2 inhibitors reduce the suppression of T cell proliferation by Mesoangioblasts/HIDEMs — CFSE labelled PBMCs were stimulated with anti CD3/CD28 beads as before in the presence of HIDEMs/mesoangioblasts and inhibitors of IDO and Cox-2, (1-Methyl-L-trypyophan (1MT) (0.5mM) and NS-398 (1.0 uM) respectively, or both. On day 6 cells were harvested and stained with anti-CD3 and 7AAD. Cells were gated on live CD3+ populations and analysed for CFSE dilution and the numbers of cells undergoing CFSE dilution were enumerated using counting beads. Experiments were carried out in duplicates. n=4. [file f1000research-2-1191-s0006.tgz › XY27FD_graph.pdf]
