# Supplementary material for: Human iPSC-derived mesoangioblasts, like their tissue-derived counterparts, suppress T cell proliferation through IDO- and PGE-2-dependent pathways
Source: F1000Res. 2013 Jan 25;2:24. [Version 1] doi: 10.12688/f1000research.2-24.v1 (PMC3968899; doi:10.12688/f1000research.2-24.v1)
Supplement: Raw data for Figure 2C: HIDEMs and mesoangioblasts fail to induce T cell proliferation in vitro — CFSE labelled PBMCs were stimulated with anti CD3/CD28 beads (PBMC+B) as a positive control. HIDEMs and mesoangioblasts were stimulated with IFN-γ, TNF-α or IL-1β (20ng/ml) for 24h. Non-stimulated or cytokine stimulated HIDEMs/mesoangioblasts (ratio 1:4) were then co-cultured with PBMC for 6 days. CD3+ CFSE labelled 7AAD- cells were enumerated using flow cytometry and counting beads. Experiments were carried out in duplicates. n=4. [file f1000research-2-1191-s0001.tgz › Immunogenicity_HIDEM_1.pdf]

	Group A	Group B	Group C	Group D	Group E	Group F	Group G	Group H	Group I
	Y	Y	Y	Y	Y	Y	Y	Y	Y
1	2543	873139	2800	4722	3882	4011	2427	4315	4614
2	3526	682448	4082	3405	3455	2902	3253	3243	3009
3	2469	756476	2721	4601	3780	3905	2355	4203	4496
4	3431	667738	3975	3312	3361	2820	3164	3154	2925
5	3664	606348	4239	3539	3590	3018	3381	3371	3129
6	2569	782967	2830	4777	3926	4056	2452	4364	4667
7	4078	670024	4705	3941	3998	3374	3770	3758	3495
8	4346	814541	5010	4202	4262	3602	4021	4008	3729

	Group J	Group K	Group L	Group M
		Data Set-K	Data Set-L	Data Set-M
	Y	Y	Y	Y
1	2000			
2	4903			
3	1938			
4	4778			
5	5089			
6	2020			
7	5631			
8	5989			
