# Supplementary material for: Human iPSC-derived mesoangioblasts, like their tissue-derived counterparts, suppress T cell proliferation through IDO- and PGE-2-dependent pathways
Source: F1000Res. 2013 Jan 25;2:24. [Version 1] doi: 10.12688/f1000research.2-24.v1 (PMC3968899; doi:10.12688/f1000research.2-24.v1)
Supplement: Raw data for Figure 2C: HIDEMs and mesoangioblasts fail to induce T cell proliferation in vitro — CFSE labelled PBMCs were stimulated with anti CD3/CD28 beads (PBMC+B) as a positive control. HIDEMs and mesoangioblasts were stimulated with IFN-γ, TNF-α or IL-1β (20ng/ml) for 24h. Non-stimulated or cytokine stimulated HIDEMs/mesoangioblasts (ratio 1:4) were then co-cultured with PBMC for 6 days. CD3+ CFSE labelled 7AAD- cells were enumerated using flow cytometry and counting beads. Experiments were carried out in duplicates. n=4. [file f1000research-2-1191-s0001.tgz › Immunogenicity_LGMD2D_Pt3.pdf]

	Group A	Group B	Group C	Group D	Group E	Group F	Group G	Group H	Group I
	Y	Y	Y	Y	Y	Y	Y	Y	Y
1	2003	503139	2900	2190	3736	4502	8494	2315	4614
2	4023	710038	3098	3495	4406	2962	1953	3243	3524
3	1940	492289	2819	2124	3637	4386	4378	2246	4496
4	3917	694734	3012	3401	4292	2879	1892	3154	3429
5	4178	734903	3221	3632	4575	3080	2036	3371	3662
6	2023	509533	2932	2212	3778	4554	4546	2339	4667
7	4639	801150	3595	4043	5071	3442	1303	3758	4076
8	4939	847466	3836	4309	5396	3673	2469	4008	4344

	Group J	Group K	Group L	Group M
		Data Set-K	Data Set-L	Data Set-M
	Y	Y	Y	Y
1	2990			
2	2303			
3	2906			
4	2234			
5	2398			
6	3022			
7	2698			
8	2886			
