# Supplementary material for: Human iPSC-derived mesoangioblasts, like their tissue-derived counterparts, suppress T cell proliferation through IDO- and PGE-2-dependent pathways
Source: F1000Res. 2013 Jan 25;2:24. [Version 1] doi: 10.12688/f1000research.2-24.v1 (PMC3968899; doi:10.12688/f1000research.2-24.v1)
Supplement: Raw data for Figure 2C: HIDEMs and mesoangioblasts fail to induce T cell proliferation in vitro — CFSE labelled PBMCs were stimulated with anti CD3/CD28 beads (PBMC+B) as a positive control. HIDEMs and mesoangioblasts were stimulated with IFN-γ, TNF-α or IL-1β (20ng/ml) for 24h. Non-stimulated or cytokine stimulated HIDEMs/mesoangioblasts (ratio 1:4) were then co-cultured with PBMC for 6 days. CD3+ CFSE labelled 7AAD- cells were enumerated using flow cytometry and counting beads. Experiments were carried out in duplicates. n=4. [file f1000research-2-1191-s0001.tgz › Immunogenicity_XY24TL.pdf]

	Group A	Group B	Group C	Group D	Group E	Group F	Group G	Group H	Group I
	Y	Y	Y	Y	Y	Y	Y	Y	Y
1	3925	498448	2082	3097	2706	3363	3753	2243	3034
2	2043	679139	2890	1922	3789	2552	3010	2789	2614
3	3821	487699	2018	3011	2629	3271	3653	2175	2949
4	1979	664500	2809	1861	3689	2478	2926	2710	2539
5	2128	702923	3006	2004	3936	2656	3130	2901	2720
6	3969	504782	2103	3131	2735	3400	3795	2266	3067
7	2404	766290	3361	2268	4375	2979	3496	3246	3049
8	2576	810592	3588	2432	4661	3184	3730	3467	3258

	Group J	Group K	Group L	Group M
		Data Set-K	Data Set-L	Data Set-M
	Y	Y	Y	Y
1	2703			
2	3690			
3	2625			
4	3591			
5	3834			
6	2732			
7	4263			
8	4542			
