# Supplementary material for: Human iPSC-derived mesoangioblasts, like their tissue-derived counterparts, suppress T cell proliferation through IDO- and PGE-2-dependent pathways
Source: F1000Res. 2013 Jan 25;2:24. [Version 1] doi: 10.12688/f1000research.2-24.v1 (PMC3968899; doi:10.12688/f1000research.2-24.v1)
Supplement: Raw data for Figure 2C: HIDEMs and mesoangioblasts fail to induce T cell proliferation in vitro — CFSE labelled PBMCs were stimulated with anti CD3/CD28 beads (PBMC+B) as a positive control. HIDEMs and mesoangioblasts were stimulated with IFN-γ, TNF-α or IL-1β (20ng/ml) for 24h. Non-stimulated or cytokine stimulated HIDEMs/mesoangioblasts (ratio 1:4) were then co-cultured with PBMC for 6 days. CD3+ CFSE labelled 7AAD- cells were enumerated using flow cytometry and counting beads. Experiments were carried out in duplicates. n=4. [file f1000research-2-1191-s0001.tgz › Immunogenicity_XY27FD.pdf]

	Group A	Group B	Group C	Group D	Group E	Group F	Group G	Group H	Group I
	Y	Y	Y	Y	Y	Y	Y	Y	Y
1	2003	593139	2990	3190	2936	4932	3494	3115	504
2	2893	709084	1998	3445	3786	2212	2239	3243	3024
3	1940	580351	2907	3102	2854	4807	3399	3029	474
4	2812	693800	1936	3352	3685	2145	2172	3154	2940
5	3009	733916	2082	3580	3933	2304	2332	3371	3144
6	2023	600678	3023	3225	2968	4989	3533	3149	505
7	3364	800074	2354	3986	4371	2596	2626	3758	3511
8	3591	846327	2523	4250	4657	2778	2811	4008	3747

	Group J	Group K	Group L	Group M
		Data Set-K	Data Set-L	Data Set-M
	Y	Y	Y	Y
1	4990			
2	2303			
3	4863			
4	2234			
5	2398			
6	5048			
7	2698			
8	2886			
