# Supplementary material for: Human iPSC-derived mesoangioblasts, like their tissue-derived counterparts, suppress T cell proliferation through IDO- and PGE-2-dependent pathways
Source: F1000Res. 2013 Jan 25;2:24. [Version 1] doi: 10.12688/f1000research.2-24.v1 (PMC3968899; doi:10.12688/f1000research.2-24.v1)
Supplement: Raw data for Figure 3B: Mesoangioblasts and HIDEMs suppress T cell proliferation in a dose dependent manner — CFSE labelled PBMCs (5 x 10^4^/well) were stimulated with anti CD3/CD28 beads (1 x 10^4^/well) (P+B) in the presence or absence of HIDEMs/mesoangioblasts at decreasing ratios (HIDEM/mesoangioblast:PBMC). On day 6 cells were harvested and stained with anti-CD3 antibody and 7AAD, and analysed by flow cytometry. CFSE dilution was analysed on gated CD3+ 7AAD- cells. The percentage of CD3+CFSE dividing cells was calculated for each group and compared to the positive control (P+B), followed by plotting against HIDEM/mesoangioblast:PBMC ratios. Experiments were carried out in duplicates. n=2 [file f1000research-2-1191-s0002.tgz › HIDEMs.pdf]

Table format: **Grouped**		Group A				Group B			
		Data Set-A				Data Set-B			
		**A:Y1**	**A:Y2**	**A:Y3**	**A:Y4**	**B:Y1**	**B:Y2**	**B:Y3**	**B:Y4**
**1**	1:4								
**2**	1:8								
**3**	1:16								
**4**	1:32								

	Group C				Group D				
	XY24TL				XY27FD				
	**C:Y1**	**C:Y2**	**C:Y3**	**C:Y4**	**D:Y1**	**D:Y2**	**D:Y3**	**D:Y4**	**E:Y1**
**1**	7.1	12.6	5.79	15.62	10.2	23.6	8.58	28.82	3.0
**2**	20.6	24.8	17.94	30.26	24.0	29.4	21.00	35.78	13.0
**3**	48.7	50.0	43.23	60.50	41.0	56.0	36.30	67.70	34.2
**4**	63.0	68.0	56.10	82.10	59.0	67.0	52.50	80.90	55.0

	Group E			Group F			
	HIDEM 1			LGMD2D Pt.9			
	**E:Y2**	**E:Y3**	**E:Y4**	**F:Y1**	**F:Y2**	**F:Y3**	**F:Y4**
**1**	5.3	2.10	6.86	1.0	4.0		
**2**	6.0	11.10	7.70	18.0	13.0		
**3**	46.0	30.18	55.70	39.0	38.1		
**4**	53.0	48.90	64.10	50.0	59.0		
