# Supplementary material for: Human iPSC-derived mesoangioblasts, like their tissue-derived counterparts, suppress T cell proliferation through IDO- and PGE-2-dependent pathways
Source: F1000Res. 2013 Jan 25;2:24. [Version 1] doi: 10.12688/f1000research.2-24.v1 (PMC3968899; doi:10.12688/f1000research.2-24.v1)
Supplement: Raw data for Figure 3C: Mesoangioblasts and HIDEMs do not interfer with T cell activation — CFSE labelled PBMCs (5 x 10^4^/well) were stimulated with anti CD3/CD28 beads (1 x 10^4^/well) (P+B) in the presence or absence of HIDEMs/mesoangioblasts at HIDEM/mesoangioblast:PBMC = 1:4 ratio. Cells were harvested on day 3, 4, 5 or 6 and analysed for CFSE dilution and expression of CD25 and CD69. The number of CD3+7AAD- cells expressing CD25 or CD69 using counting beads and the % of CD25+ and CD69+ cells were calculated from the data. Experiments were carried out in duplicates. n=2. [file f1000research-2-1191-s0003.tgz › __of_CD25_expressing_T_cells.pdf]

Table format: **Grouped**		Group A				Group B			
		P				P+B			
		**A:Y1**	**A:Y2**	**A:Y3**	**A:Y4**	**B:Y1**	**B:Y2**	**B:Y3**	**B:Y4**
**1**	Day 3	3.1	5.2	2.6	6.3	14.0	18.0	12.5	21.0
**2**	Day 4	5.3	6.0	4.6	7.2	67.0	60.1	60.7	69.4
**3**	Day 5	4.4	3.9	3.8	4.8	59.0	53.2	53.4	61.5
**4**	Day 6	3.9	4.4	3.3	5.4	50.0	52.1	45.3	60.2

	Group C				Group D				
	XY24TL				XY27FD				
	C:Y1	C:Y2	C:Y3	C:Y4	D:Y1	D:Y2	D:Y3	D:Y4	E:Y1
1	6.0	8.1	5.3	9.6	12.0	13.0	10.7	15.3	13.0
2	69.0	76.0	62.5	87.7	65.0	70.0	58.9	80.8	67.0
3	59.0	67.0	53.4	77.4	55.0	69.4	49.8	80.1	60.0
4	52.0	58.0	47.1	67.0	62.1	50.0	56.3	57.8	65.0

	Group E			Group F			
	HIDEM 1			LGMD2D Pt.3			
	**E:Y2**	**E:Y3**	**E:Y4**	**F:Y1**	**F:Y2**	**F:Y3**	**F:Y4**
**1**	10.0	11.6	11.8	9.0	11.9	8.0	14.0
**2**	72.0	60.7	83.1	66.0	75.0	59.8	86.6
**3**	58.0	54.3	67.0	60.8	62.6	55.1	72.3
**4**	51.0	58.9	59.0	56.0	63.0	50.7	72.8
