# Supplementary material for: Human iPSC-derived mesoangioblasts, like their tissue-derived counterparts, suppress T cell proliferation through IDO- and PGE-2-dependent pathways
Source: F1000Res. 2013 Jan 25;2:24. [Version 1] doi: 10.12688/f1000research.2-24.v1 (PMC3968899; doi:10.12688/f1000research.2-24.v1)
Supplement: Raw data for Figure 3C: Mesoangioblasts and HIDEMs do not interfer with T cell activation — CFSE labelled PBMCs (5 x 10^4^/well) were stimulated with anti CD3/CD28 beads (1 x 10^4^/well) (P+B) in the presence or absence of HIDEMs/mesoangioblasts at HIDEM/mesoangioblast:PBMC = 1:4 ratio. Cells were harvested on day 3, 4, 5 or 6 and analysed for CFSE dilution and expression of CD25 and CD69. The number of CD3+7AAD- cells expressing CD25 or CD69 using counting beads and the % of CD25+ and CD69+ cells were calculated from the data. Experiments were carried out in duplicates. n=2. [file f1000research-2-1191-s0003.tgz › __of_CD69_expressing_T_cells.pdf]

Table format: **Grouped**		Group A				Group B			
		P				P+B			
		**A:Y1**	**A:Y2**	**A:Y3**	**A:Y4**	**B:Y1**	**B:Y2**	**B:Y3**	**B:Y4**
**1**	Day 3	2.3	3.0	1.9	3.8	22.1	26.4	19.9	30.7
**2**	Day 4	2.1	1.2	1.7	0.3	37.7	35.0	34.1	38.0
**3**	Day 5	1.9	2.9	1.5	3.6	28.3	28.9	25.5	33.5
**4**	Day 6	3.4	2.5	2.9	3.2	23.9	25.1	21.5	29.2

	Group C				Group D				
	XY24TL				XY27FD				
	C:Y1	C:Y2	C:Y3	C:Y4	D:Y1	D:Y2	D:Y3	D:Y4	E:Y1
1	5.7	7.8	5.0	9.3	17.2	13.5	15.4	15.8	20.0
2	36.2	29.6	32.7	34.3	41.6	32.4	37.6	37.6	34.5
3	31.8	32.3	28.7	37.4	32.4	31.1	29.3	36.1	30.0
4	27.2	26.5	24.5	30.8	26.7	27.6	24.1	32.0	23.7

	Group E			Group F			
	HIDEM 1			LGMD2D Pt.3			
	**E:Y2**	**E:Y3**	**E:Y4**	**F:Y1**	**F:Y2**	**F:Y3**	**F:Y4**
**1**	17.5	18.0	20.4	15.2	15.9	13.6	18.6
**2**	40.5	31.2	46.9	38.9	39.9	35.2	46.2
**3**	30.6	27.1	35.5	31.5	27.6	28.4	32.0
**4**	25.3	21.3	29.4	21.0	28.6	18.9	33.2
