# Supplementary material for: Human iPSC-derived mesoangioblasts, like their tissue-derived counterparts, suppress T cell proliferation through IDO- and PGE-2-dependent pathways
Source: F1000Res. 2013 Jan 25;2:24. [Version 1] doi: 10.12688/f1000research.2-24.v1 (PMC3968899; doi:10.12688/f1000research.2-24.v1)
Supplement: Raw data for Figure 4A: Neutralising antibodies against IFN-γ and TNF-α reduce the immunosuppressive capacity of Mesoangioblasts/HIDEMs — CFSE labelled PBMCs were stimulated with anti-CD3/CD28 beads in the presence of HIDEMs/mesoangioblasts (1:4) and neutralising antibodies against IFN-γ and TNF-α or irrelevant isotype control antibody (0.5, 1.0 and 2.0 µg/ml) or recombinant IL-1RA (0.5, 1.0 and 2.0 µg/ml). Cells were harvested on day 6 and stained with anti-CD3 and 7AAD. After gating on CD3+7AAD- the number of CFSE diluting cells were enumerated using counting beads. Experiments were carried out in duplicates. n=4. [file f1000research-2-1191-s0004.tgz › HIDEM_1.pdf]

	Group A	Group B	Group C	Group D	Group E	Group F
	Y	Y	Y	Y	Y	Y
1	3008	1715614	185615	193170	742924	935843
2	9172	1629552	197774	230974	1249544	2179774
3	3509	1614397	217920	226791	1107129	898814
4	10747	1913345	232197	271179	1867152	1972314
5	8643	1633273	186100	217338	1175720	980528
6	2431	1389943	150374	156496	763928	758191
7	7425	1320218	160225	187123	812344	1060906
8	5973	1657968	128419	149973	611257	590574

	Group G	Group H	Group I	Group J	Group K	Group L
				Data Set-J	Data Set-K	Data Set-L
	**Y**	**Y**	**Y**	**Y**	**Y**	**Y**
**1**	634840	1179867	380581	113586	200016	217705
**2**	186102	1091891	353954	190285	111463	106952
**3**	745386	1385339	446843	133346	234829	255600
**4**	218492	1282041	415579	223404	130853	125557
**5**	175118	1027383	333052	179054	104890	100645
**6**	514326	955894	308331	92018	162041	176373
**7**	150769	884618	286759	154158	90298	86644
**8**	120841	708904	229815	123557	72383	69455

	Group M	Group N	Group O
	Y	Y	Y
1	113586	260016	127705
2	100285	111463	106952
3	133346	305279	149925
4	117729	130853	125557
5	94372	104890	100645
6	92018	210652	103458
7	81242	90298	86644
8	65126	72383	69455
