# Supplementary material for: Human iPSC-derived mesoangioblasts, like their tissue-derived counterparts, suppress T cell proliferation through IDO- and PGE-2-dependent pathways
Source: F1000Res. 2013 Jan 25;2:24. [Version 1] doi: 10.12688/f1000research.2-24.v1 (PMC3968899; doi:10.12688/f1000research.2-24.v1)
Supplement: Raw data for Figure 4A: Neutralising antibodies against IFN-γ and TNF-α reduce the immunosuppressive capacity of Mesoangioblasts/HIDEMs — CFSE labelled PBMCs were stimulated with anti-CD3/CD28 beads in the presence of HIDEMs/mesoangioblasts (1:4) and neutralising antibodies against IFN-γ and TNF-α or irrelevant isotype control antibody (0.5, 1.0 and 2.0 µg/ml) or recombinant IL-1RA (0.5, 1.0 and 2.0 µg/ml). Cells were harvested on day 6 and stained with anti-CD3 and 7AAD. After gating on CD3+7AAD- the number of CFSE diluting cells were enumerated using counting beads. Experiments were carried out in duplicates. n=4. [file f1000research-2-1191-s0004.tgz › LGMD2D_Pt3.pdf]

	Group A	Group B	Group C	Group D	Group E	Group F
	Y	Y	Y	Y	Y	Y
1	3401	1421121	203052	371265	958768	969718
2	7761	1335879	240515	432679	1200882	645948
3	3970	1668612	238395	435904	1125732	1138589
4	9090	1568524	282382	508015	1410015	758429
5	7315	1256954	226316	407124	1129934	607791
6	2749	1151352	164502	300784	776765	785636
7	6281	1082291	194853	350540	972920	523325
8	5057	867308	156167	280925	779664	419386

	Group G	Group H	Group I	Group J	Group K	Group L
				Data Set-J	Data Set-K	Data Set-L
	Y	Y	Y	Y	Y	Y
1	169144	314267	397086	287316	259193	272731
2	244147	395142	475275	344266	304750	286821
3	198581	368980	466223	337334	304313	320209
4	286646	463940	558030	404203	357804	336752
5	229733	371805	447204	323936	286755	269885
6	137030	254606	321703	232770	209986	220954
7	197795	320128	385050	278909	246894	232369
8	158525	256555	308580	223525	197870	186230

	Group M	Group N	Group O
	Y	Y	Y
1	287316	159193	172731
2	244266	304750	286821
3	337334	186896	202792
4	286786	357804	336752
5	229845	286755	269885
6	232770	128968	139936
7	197892	246894	232369
8	158602	197870	186230
