# Supplementary material for: Human iPSC-derived mesoangioblasts, like their tissue-derived counterparts, suppress T cell proliferation through IDO- and PGE-2-dependent pathways
Source: F1000Res. 2013 Jan 25;2:24. [Version 1] doi: 10.12688/f1000research.2-24.v1 (PMC3968899; doi:10.12688/f1000research.2-24.v1)
Supplement: Raw data for Figure 4A: Neutralising antibodies against IFN-γ and TNF-α reduce the immunosuppressive capacity of Mesoangioblasts/HIDEMs — CFSE labelled PBMCs were stimulated with anti-CD3/CD28 beads in the presence of HIDEMs/mesoangioblasts (1:4) and neutralising antibodies against IFN-γ and TNF-α or irrelevant isotype control antibody (0.5, 1.0 and 2.0 µg/ml) or recombinant IL-1RA (0.5, 1.0 and 2.0 µg/ml). Cells were harvested on day 6 and stained with anti-CD3 and 7AAD. After gating on CD3+7AAD- the number of CFSE diluting cells were enumerated using counting beads. Experiments were carried out in duplicates. n=4. [file f1000research-2-1191-s0004.tgz › XY24TL.pdf]

	Group A	Group B	Group C	Group D	Group E	Group F
	Y	Y	Y	Y	Y	Y
1	2885	1162139	190274	605234	919231	1265555
2	7717	1538949	314892	575777	1302252	824574
3	3364	1364524	223391	710624	1079309	1485951
4	9038	3159213	369713	676036	1529040	968166
5	7274	1430297	296297	541767	1225314	775862
6	2331	941531	154149	490340	744733	1025316
7	6246	1489866	255111	466475	1055047	668044
8	5029	1193914	204455	373829	845476	535354

	Group G	Group H	Group I	Group J	Group K	Group L
				Data Set-J	Data Set-K	Data Set-L
	Y	Y	Y	Y	Y	Y
1	189126	212245	377087	312245	399238	176480
2	115550	468844	518956	468844	332272	483585
3	222043	249188	442741	366605	468749	207195
4	135652	550478	609319	550478	390121	567787
5	108735	441152	488304	441152	312651	455022
6	153219	171949	305501	252967	323446	142974
7	93610	379839	420440	379839	269193	391782
8	75037	304405	336939	304405	215739	313975

	Group M	Group N	Group O
	Y	Y	Y
1	399238	176480	251825
2	222272	183585	161380
3	468749	207195	295662
4	260962	215536	189464
5	209151	172749	151857
6	323446	142974	204016
7	180073	148729	130740
8	144324	119207	104791
