# Supplementary material for: Human iPSC-derived mesoangioblasts, like their tissue-derived counterparts, suppress T cell proliferation through IDO- and PGE-2-dependent pathways
Source: F1000Res. 2013 Jan 25;2:24. [Version 1] doi: 10.12688/f1000research.2-24.v1 (PMC3968899; doi:10.12688/f1000research.2-24.v1)
Supplement: Raw data for Figure 4A: Neutralising antibodies against IFN-γ and TNF-α reduce the immunosuppressive capacity of Mesoangioblasts/HIDEMs — CFSE labelled PBMCs were stimulated with anti-CD3/CD28 beads in the presence of HIDEMs/mesoangioblasts (1:4) and neutralising antibodies against IFN-γ and TNF-α or irrelevant isotype control antibody (0.5, 1.0 and 2.0 µg/ml) or recombinant IL-1RA (0.5, 1.0 and 2.0 µg/ml). Cells were harvested on day 6 and stained with anti-CD3 and 7AAD. After gating on CD3+7AAD- the number of CFSE diluting cells were enumerated using counting beads. Experiments were carried out in duplicates. n=4. [file f1000research-2-1191-s0004.tgz › XY27FD.pdf]

	Group A	Group B	Group C	Group D	Group E	Group F
	Y	Y	Y	Y	Y	Y
1	2806	1151252	212127	413593	341372	557038
2	9124	1329764	350702	393559	593914	2062737
3	3271	1351741	249049	485605	2153056	654033
4	10690	1561344	411760	462081	2871500	247809
5	8598	1251200	329991	370316	499741	1999952
6	2267	932711	171854	335077	319618	451293
7	7385	1077337	284124	318846	1991345	460998
8	5942	863338	227704	255528	534831	689976

	Group G	Group H	Group I	Group J	Group K	Group L
				Data Set-J	Data Set-K	Data Set-L
	Y	Y	Y	Y	Y	Y
1	144423	160799	393438	415599	202248	405183
2	238186	916716	539222	233750	535190	357187
3	169553	188782	461939	487960	237450	475730
4	279648	206688	633114	274439	628380	419374
5	224124	286196	507372	219950	503578	336093
6	117001	130269	318748	336702	163850	328263
7	192966	218625	436858	189372	433592	289378
8	154655	335485	350096	151775	347478	231914

	Group M	Group N	Group O
	Y	Y	Y
1	415599	202248	405183
2	333750	435190	257187
3	487960	237450	475730
4	391855	510963	301957
5	314041	409487	242002
6	336702	163850	328263
7	270390	352574	208360
8	216698	282556	166991
