# Supplementary material for: Human iPSC-derived mesoangioblasts, like their tissue-derived counterparts, suppress T cell proliferation through IDO- and PGE-2-dependent pathways
Source: F1000Res. 2013 Jan 25;2:24. [Version 1] doi: 10.12688/f1000research.2-24.v1 (PMC3968899; doi:10.12688/f1000research.2-24.v1)
Supplement: Raw data for Figure 4B: Pre-stimulation with IFN-γ, TNF-α and IL-1β does not enhance the immunosuppressive effect of Mesoangioblasts/HIDEMs — HIDEMs/mesoangioblasts were left untreated or were stimulated with IFN-γ, TNF-α or IL-1β (20ng/ml) for 24h before setting up co-cultures with CFSE labelled PBMC and anti CD3/CD28 beads. After 6 days cells were harvested and surface stained for CD3 and 7AAD before analysis of CFSE dilution. CD3+CFSE diluted cell numbers were calculated using counting beads as before. Experiments were carried out in duplicates. n=4. [file f1000research-2-1191-s0005.tgz › HIDEM_1.pdf]

Table format: **Column**		Group A	Group B	Group C	Group D	Group E	Group F	Group G	Group H
		**Y**	**Y**	**Y**	**Y**	**Y**	**Y**	**Y**	**Y**
**1**	2334.627	2272	465512	48542	84892	96020	89145	99151	108600
**2**	2081.865	2557	614864	71191	94997	68579	55264	74330	157881
**3**	Title	2644	446566	56973	99655	112721	104648	116396	127492
**4**	Title	2979	721931	83568	111519	80501	64866	87253	155356
**5**	Title	2419	878544	66998	89396	64540	52011	69951	108565
**6**	Title	1834	377140	39321	68771	77787	72216	80323	87979
**7**	Title	2065	498142	57671	76958	55555	44767	60214	107905
**8**	Title	1678	399205	46238	61693	44542	35897	48276	72519

	Group I	Group J
	Y	Y
1	100090	155211
2	109637	123454
3	87499	152221
4	222643	144933
5	78444	116172
6	81084	125742
7	53633	100013
8	183136	80168
