# Supplementary material for: Human iPSC-derived mesoangioblasts, like their tissue-derived counterparts, suppress T cell proliferation through IDO- and PGE-2-dependent pathways
Source: F1000Res. 2013 Jan 25;2:24. [Version 1] doi: 10.12688/f1000research.2-24.v1 (PMC3968899; doi:10.12688/f1000research.2-24.v1)
Supplement: Raw data for Figure 4B: Pre-stimulation with IFN-γ, TNF-α and IL-1β does not enhance the immunosuppressive effect of Mesoangioblasts/HIDEMs — HIDEMs/mesoangioblasts were left untreated or were stimulated with IFN-γ, TNF-α or IL-1β (20ng/ml) for 24h before setting up co-cultures with CFSE labelled PBMC and anti CD3/CD28 beads. After 6 days cells were harvested and surface stained for CD3 and 7AAD before analysis of CFSE dilution. CD3+CFSE diluted cell numbers were calculated using counting beads as before. Experiments were carried out in duplicates. n=4. [file f1000research-2-1191-s0005.tgz › LGMD2D_Pt3.pdf]

Table format: **Column**		Group A	Group B	Group C	Group D	Group E	Group F	Group G	Group H
		Y	Y	Y	Y	Y	Y	Y	Y
**1**	2334.627	2715	1618679	147326	366936	343434	209486	292440	108284
**2**	2081.865	10880	1053345	119011	79039	86849	265296	170582	238230
**3**	Title	3164	2900579	172962	830822	803226	245949	343351	127121
**4**	Title	12752	1236781	139716	92782	101953	311479	200269	279699
**5**	Title	10250	991115	111992	74381	81730	249632	160515	224166
**6**	Title	2193	1311409	119353	297277	278236	169714	236921	87723
**7**	Title	8808	853389	96414	64029	70357	214930	138195	193002
**8**	Title	7082	683879	77284	51333	56403	172256	110765	154684

	Group I	Group J
	Y	Y
1	196932	334387
2	156043	245163
3	231208	392603
4	183197	287840
5	146835	230689
6	159543	270906
7	126416	198619
8	101326	159185
