# Supplementary material for: Human iPSC-derived mesoangioblasts, like their tissue-derived counterparts, suppress T cell proliferation through IDO- and PGE-2-dependent pathways
Source: F1000Res. 2013 Jan 25;2:24. [Version 1] doi: 10.12688/f1000research.2-24.v1 (PMC3968899; doi:10.12688/f1000research.2-24.v1)
Supplement: Raw data for Figure 4B: Pre-stimulation with IFN-γ, TNF-α and IL-1β does not enhance the immunosuppressive effect of Mesoangioblasts/HIDEMs — HIDEMs/mesoangioblasts were left untreated or were stimulated with IFN-γ, TNF-α or IL-1β (20ng/ml) for 24h before setting up co-cultures with CFSE labelled PBMC and anti CD3/CD28 beads. After 6 days cells were harvested and surface stained for CD3 and 7AAD before analysis of CFSE dilution. CD3+CFSE diluted cell numbers were calculated using counting beads as before. Experiments were carried out in duplicates. n=4. [file f1000research-2-1191-s0005.tgz › XY24TL.pdf]

Table format: **Column**		Group A	Group B	Group C	Group D	Group E	Group F	Group G	Group H
		**Y**	**Y**	**Y**	**Y**	**Y**	**Y**	**Y**	**Y**
**1**	2334.627	2335	484517	31180	36100	27058	24081	23560	43851
**2**	2081.865	2082	608296	47178	52526	102583	65844	66462	99463
**3**	Title	2718	568881	36588	42364	31747	28252	27640	51465
**4**	Title	2421	714219	55372	61652	120426	77289	78014	116763
**5**	Title	1972	572364	44403	49435	96534	61966	62548	93598
**6**	Title	1885	692537	25255	29241	21915	19503	19081	35521
**7**	Title	1680	492821	38216	42549	83104	53339	53839	80576
**8**	Title	1370	394941	30648	34120	66618	42766	43167	64592

	Group I	Group J
	Y	Y
1	34248	117814
2	76593	59042
3	40190	138310
4	89910	69302
5	72080	55566
6	27741	95443
7	62048	47828
8	49745	38350
