# Supplementary material for: Human iPSC-derived mesoangioblasts, like their tissue-derived counterparts, suppress T cell proliferation through IDO- and PGE-2-dependent pathways
Source: F1000Res. 2013 Jan 25;2:24. [Version 1] doi: 10.12688/f1000research.2-24.v1 (PMC3968899; doi:10.12688/f1000research.2-24.v1)
Supplement: Raw data for Figure 4B: Pre-stimulation with IFN-γ, TNF-α and IL-1β does not enhance the immunosuppressive effect of Mesoangioblasts/HIDEMs — HIDEMs/mesoangioblasts were left untreated or were stimulated with IFN-γ, TNF-α or IL-1β (20ng/ml) for 24h before setting up co-cultures with CFSE labelled PBMC and anti CD3/CD28 beads. After 6 days cells were harvested and surface stained for CD3 and 7AAD before analysis of CFSE dilution. CD3+CFSE diluted cell numbers were calculated using counting beads as before. Experiments were carried out in duplicates. n=4. [file f1000research-2-1191-s0005.tgz › XY27FD.pdf]

Table format: **Column**		Group A	Group B	Group C	Group D	Group E	Group F	Group G	Group H
		**Y**	**Y**	**Y**	**Y**	**Y**	**Y**	**Y**	**Y**
**1**	2334.627	3209	462880	73510	94298	54238	70637	91118	177670
**2**	2081.865	2203	457227	58538	108691	67240	145131	134719	90577
**3**	Title	3745	443476	86290	110699	63662	82917	106965	208591
**4**	Title	2564	1606506	68711	127598	78928	170385	158160	106329
**5**	Title	2086	406586	55092	102281	63279	136568	126771	85237
**6**	Title	2594	475008	59550	76392	43936	57222	73815	143938
**7**	Title	1779	394499	47420	88052	54470	117575	109140	73377
**8**	Title	1449	1256554	38023	70583	43672	94241	87482	58823

	Group I	Group J
	Y	Y
1	147021	121559
2	104798	217190
3	172604	142707
4	123027	254994
5	98618	204369
6	119107	98478
7	84898	175955
8	68056	141024
