# Supplementary material for: Human iPSC-derived mesoangioblasts, like their tissue-derived counterparts, suppress T cell proliferation through IDO- and PGE-2-dependent pathways
Source: F1000Res. 2013 Jan 25;2:24. [Version 1] doi: 10.12688/f1000research.2-24.v1 (PMC3968899; doi:10.12688/f1000research.2-24.v1)
Supplement: Raw data for Figure 5: The presence of IDO and PGE-2 inhibitors reduce the suppression of T cell proliferation by Mesoangioblasts/HIDEMs — CFSE labelled PBMCs were stimulated with anti CD3/CD28 beads as before in the presence of HIDEMs/mesoangioblasts and inhibitors of IDO and Cox-2, (1-Methyl-L-trypyophan (1MT) (0.5mM) and NS-398 (1.0 uM) respectively, or both. On day 6 cells were harvested and stained with anti-CD3 and 7AAD. Cells were gated on live CD3+ populations and analysed for CFSE dilution and the numbers of cells undergoing CFSE dilution were enumerated using counting beads. Experiments were carried out in duplicates. n=4. [file f1000research-2-1191-s0006.tgz › HIDEM_1.pdf]

	Group A	Group B	Group C	Group D	Group E	Group F	Group G	Group H	Group I
							Data Set-G	Data Set-H	Data Set-I
	**Y**	**Y**	**Y**	**Y**	**Y**	**Y**	**Y**	**Y**	**Y**
**1**	3535	1892033	128643	1089341	372379	1502145			
**2**	2548	1777784	111111	629119	447155	1030962			
**3**	4745	1595858	115199	904672	388902	1380914			
**4**	1804	1482698	99433	716278	468012	1183553			
**5**	4544	835727	64624	421169	251505	693060			
**6**	9834	800360	69809	332671	367105	528126			
**7**	4745	1195858	136083	334650	206567	993303			
**8**	10084	1082698	114584	551667	188231	544434			
