# Supplementary material for: Human iPSC-derived mesoangioblasts, like their tissue-derived counterparts, suppress T cell proliferation through IDO- and PGE-2-dependent pathways
Source: F1000Res. 2013 Jan 25;2:24. [Version 1] doi: 10.12688/f1000research.2-24.v1 (PMC3968899; doi:10.12688/f1000research.2-24.v1)
Supplement: Raw data for Figure 5: The presence of IDO and PGE-2 inhibitors reduce the suppression of T cell proliferation by Mesoangioblasts/HIDEMs — CFSE labelled PBMCs were stimulated with anti CD3/CD28 beads as before in the presence of HIDEMs/mesoangioblasts and inhibitors of IDO and Cox-2, (1-Methyl-L-trypyophan (1MT) (0.5mM) and NS-398 (1.0 uM) respectively, or both. On day 6 cells were harvested and stained with anti-CD3 and 7AAD. Cells were gated on live CD3+ populations and analysed for CFSE dilution and the numbers of cells undergoing CFSE dilution were enumerated using counting beads. Experiments were carried out in duplicates. n=4. [file f1000research-2-1191-s0006.tgz › LGMD2D_Pt3.pdf]

	Group A	Group B	Group C	Group D	Group E	Group F	Group G	Group H	Group I
							Data Set-G	Data Set-H	Data Set-I
	Y	Y	Y	Y	Y	Y	Y	Y	Y
1	3061	1691058	143593	1348372	856763	1307296			
2	3375	1475709	124097	1101259	427440	1898611			
3	4745	995858	115199	604672	388902	980914			
4	1804	882698	299433	416278	468012	883553			
5	4788	1691616	438135	1171859	472170	1093060			
6	6680	1075142	347195	813856	587818	1528126			
7	1673	1135115	122428	627934	328976	723231			
8	3379	1028156	103781	375533	304035	417945			
