# Supplementary material for: Human iPSC-derived mesoangioblasts, like their tissue-derived counterparts, suppress T cell proliferation through IDO- and PGE-2-dependent pathways
Source: F1000Res. 2013 Jan 25;2:24. [Version 1] doi: 10.12688/f1000research.2-24.v1 (PMC3968899; doi:10.12688/f1000research.2-24.v1)
Supplement: Raw data for Figure 5: The presence of IDO and PGE-2 inhibitors reduce the suppression of T cell proliferation by Mesoangioblasts/HIDEMs — CFSE labelled PBMCs were stimulated with anti CD3/CD28 beads as before in the presence of HIDEMs/mesoangioblasts and inhibitors of IDO and Cox-2, (1-Methyl-L-trypyophan (1MT) (0.5mM) and NS-398 (1.0 uM) respectively, or both. On day 6 cells were harvested and stained with anti-CD3 and 7AAD. Cells were gated on live CD3+ populations and analysed for CFSE dilution and the numbers of cells undergoing CFSE dilution were enumerated using counting beads. Experiments were carried out in duplicates. n=4. [file f1000research-2-1191-s0006.tgz › XY24TL.pdf]

	Group A	Group B	Group C	Group D	Group E	Group F	Group G	Group H	Group I
							Data Set-G	Data Set-H	Data Set-I
	Y	Y	Y	Y	Y	Y	Y	Y	Y
1	2273	854032	105921	345865	203973	848357			
2	7637	1108323	109209	384845	260978	458183			
3	4166	1724736	280512	969418	368728	1014850			
4	8098	2071022	334316	688613	597014	1811167			
5	4544	835727	289400	633805	230088	520850			
6	9834	800360	146764	493526	365814	979864			
7	4745	1595858	395131	907363	449038	1222956			
8	180084	1482698	329248	786952	741406	1067947			
