# Supplementary material for: Human iPSC-derived mesoangioblasts, like their tissue-derived counterparts, suppress T cell proliferation through IDO- and PGE-2-dependent pathways
Source: F1000Res. 2013 Jan 25;2:24. [Version 1] doi: 10.12688/f1000research.2-24.v1 (PMC3968899; doi:10.12688/f1000research.2-24.v1)
Supplement: Peer review history

# Peer review: Referee response for version 1

**Published:** 2014-02-24

**Authors:** Mayana Zatz, Antonio Condino-Neto

**Affiliations:** 1 Human Genome and Stem-Cell Center, Institute of Biosciences, University of São Paulo, Sao Pãulo, Brazil; 2 Department of Immunology, Institute of Biomedical Sciences, University of São Paulo, São Paulo, Brazil

In this study Li, English *et al.* propose that HIDEMs behave in a very similar manner to mesoangioblasts, with regard to expression of HLA and co-stimulatory molecules, suppressive potency and mechanisms of action. HIDEMs are comparable to mesoangioblasts in their surface marker expression and that both cell populations respond to stimulation with pro-inflammatory cytokines consistently, with increased expression of HLA-ABC, HLA-DR, PD-L1 and low level expression of CD40. Importantly, HIDEMs suppress T cell proliferation driven by anti-CD3/CD28 stimulation in a similar dose-dependent manner as that of mesoangioblasts, but with increased potency. Comparable to mesoangioblasts, HIDEMs did not alter the expression of early activation markers CD25 or CD69 on CD3+ T cells, a phenomenon also seen with immune-suppressive mesenchymal stromal cells (MSC). These findings are comparable to other publications by the same group.

 

The activation step involving IFN-γ or TNF-α is an apparent prerequisite for HIDEM suppression of T cell proliferation as neutralization of either cytokine significantly abolished the inhibitory effect. IDO and PGE-2 are identified as key mechanisms of action involved in HIDEM suppression of T cell proliferation.

 

Following this line of thinking we suggest that other potential suppressive mechanisms should be investigated. The involvement of regulatory cytokines like IL-10, TGF-β for example should be addressed, as well as the more detailed phenotype of T cells or even the participation of T regulatory cells should be explored.

 

The detailed study of immunological mechanisms is highly relevant and may contribute to advance our knowledge about the use of HIDEMs or mesangioblasts for the therapy of Duchenne muscular dystrophy.

We have read this submission. We believe that we have an appropriate level of expertise to confirm that it is of an acceptable scientific standard.

# Peer review: Referee response for version 1

**Published:** 2013-01-31

**Authors:** Philippe Saas

**Affiliations:** 1 Department of Immunology and Cell Therapy, University of Franche-Comté, Besançon, France

This is an interesting article reporting the *in vitro* effect of human iPSC-derived mesoangioblasts on T cells. This is a prerequisite step in order to use these cells in the treatment of patients suffering from muscular dystrophy.

How do human iPSC-derived mesoanglioblasts interact with T cells is an open question. This is of interest since these cells can be used in cell-based therapy approaches. Immunogenicity of infused cells depends on cell type.

The study by Li and colleagues is well performed. I have only minor comments in order to better explain how the results on Figure #3AB are shown or calculated. 

Typo (page 3, paragraph “Results”): “makers” instead of “markers”.

I propose to add the number of experiments performed at the end of the legend of the Figure #1.

I suggest that the authors improve the Figure #3 panel A and B and its legend. For Figure #3A, I understand well the two upper panels: the panel PBMC shows non proliferating PBMC (all the cells are brightly stained with CSFE) and the panel PBMC + Anti CD3/CD28 shows maximal proliferation with all the cells (gated for CD3) exhibiting decreased CSFE fluorescence due to CSFE dilution occurring during cell division. I propose to modify the PBMC histogram and to increase the scale to better appreciate the peak. According to the legend and the Materials and Methods section, the other panels correspond to the coculture of either mesangioblast or iPSC-derived mesangioblast from a healthy donor or a patient with PBMC (fixed number of 5 x 10 ^4^/well). Thus, the number of mesangioblasts varies and is decreasing (from 1:4 to 1:32). It is logical that the peaks of proliferating CD3+ cells increase in the lower panels. However, if inhibition of T cell activation occurs, a peak in the non dividing area/region should be observed as in the panel “PBMC”. It seems to be the case for the condition “LMGMDD pt.3”. However, this is difficult to observe with the other conditions. I propose to increase the Y scale. Maybe, as performed in the previous Figure (Figure #2), counting beads were added to assess the absolute number of CD3+ CFSE ^low^ 7AAD ^neg^ cells. This explains why the peaks are flat in the 1:4 ratio conditions. Concerning Figure #3B, the shown data represent the percentage of CD3+ cell proliferation in coculture divided by the percentage of CD3+ cell proliferation in the absence of coculture. According to the upper panel of the Figure #3A, all the cells proliferate. I suggest that the authors provide more explanations on how the data are obtained.

I have read this submission. I believe that I have an appropriate level of expertise to confirm that it is of an acceptable scientific standard.
